# An RNA-binding regulatory cascade controls the switch from proliferation to differentiation in the *Drosophila* male germ cell lineage

**DOI:** 10.1073/pnas.2418279122

**Published:** 2025-05-16

**Authors:** Devon E. Harris, Jongmin J. Kim, Sarah R. Stern, Hannah M. Vicars, Neuza R. Matias, Lorenzo Gallicchio, Catherine C. Baker, Margaret T. Fuller

**Affiliations:** ^a^Department of Developmental Biology, Stanford University School of Medicine, Stanford, CA 94305; ^b^Department of Chemical and Systems Biology, Stanford University School of Medicine, Stanford, CA 94305; ^c^Department of Genetics, Stanford University School of Medicine, Stanford, CA 94305

**Keywords:** differentiation, proliferation, spermatogonia, RNA-binding proteins, adult stem cells

## Abstract

The switch from precursor cell proliferation to onset of differentiation in adult stem cell lineages must be carefully regulated to produce sufficient progeny to maintain and repair tissues, yet prevent overproliferation that may enable oncogenesis. In the *Drosophila* male germ cell lineage, spermatogonia produced by germ line stem cells undergo a limited number of transit amplifying mitotic divisions before switching to the spermatocyte program that sets up meiosis and eventual spermatid differentiation. The number of transit amplifying divisions is set by accumulation of the *bag-of-marbles* (Bam) protein to a critical threshold. In *bam* mutants, spermatogonia proliferate through several extra rounds of mitosis and then die without becoming spermatocytes. Here, we show that a key role of Bam for the mitosis to differentiation switch is repressing expression of Held Out Wings (*how*), homolog of mammalian Quaking. Knockdown of *how* in germ cells was sufficient to allow spermatogonia mutant for *bam* or its partner *benign gonial cell neoplasm* to differentiate, while forced expression of nuclear-targeted How protein in spermatogonia wild-type for *bam* resulted in continued proliferation at the expense of differentiation. Our findings suggest that Bam targets *how* RNA for degradation by acting as an adapter to recruit the CCR4-NOT deadenylation complex via binding its subunit, Caf40. As How is itself an RNA-binding protein with roles in RNA processing, our findings reveal that the switch from proliferation to meiosis and differentiation in the *Drosophila* male germ line adult stem cell lineage is regulated by a cascade of RNA-binding proteins.

## Significance

In the stem cell lineages that maintain short-lived specialized cell types and repair many tissues in our body, progenitors proliferate but then must stop dividing and differentiate into the appropriate cell type(s). Proper regulation of the switch from proliferation to differentiation is key to ensure production of sufficient cells to maintain tissues but avoid continued precursor cell proliferation that could progress to cancer. Here, we show that the switch from proliferation to differentiation in the *Drosophila* male germ line adult stem cell lineage is governed by a cascade of RNA-binding proteins, where the differentiation factor Bag-of Marbles (Bam) down-regulates expression of the RNA-binding protein Held-Out-Wings, homolog of mammalian *Quaking*, likely by recruiting the CCR4-NOT RNA degradation complex.

In a common feature of the adult stem cell lineages that build and repair tissues throughout the body, relatively less differentiated precursor cells undergo a limited series of transit amplifying divisions, which serve to increase the number of differentiated progeny produced from a single adult stem cell division. Blood, skin, intestinal epithelia, and male germ cell lineages all employ transit amplifying (TA) divisions to produce large numbers of differentiated cells (1). In this context, the switch from proliferation to differentiation must be carefully regulated. Premature switching can lead to inadequate differentiated cell replacement, with defects in tissue maintenance or repair. Conversely, failure to switch resulting in overproliferation of precursor cells may increase danger of accumulating oncogenic mutations leading to cancer (2).

**Fig. 1.**
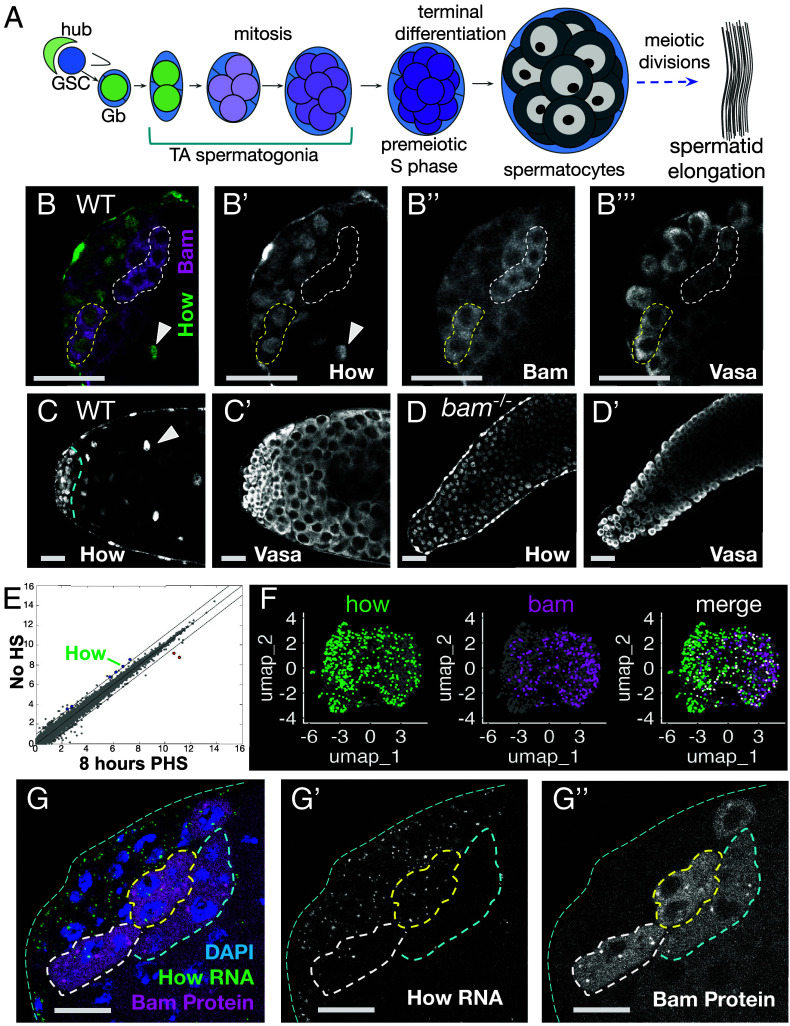
Nuclear How persists in *bam* mutant spermatogonia. (*A*) Diagram of *Drosophila* early male germ line development. (*B*–*B’’’*) Immunofluorescence images of the apical tip of a wild type testis stained with anti-How, anti-Vasa, and anti-Bam. (*B*) merge of (green) How and (magenta) Bam. (*B’*–*B’’’*) Black and white single channels showing (*B’*) How, (*B’’*) Bam, and (*B’’’*) Vasa to mark germ cells. (*C* and *D*) Immunofluorescence images of (*C*) wild type and (*D*) *bam^1/∆86^* testis apical tips stained with anti-How and anti-Vasa. The dashed cyan line in (*C*) marks the border between spermatogonia (left of line) and the spermatocyte region (*Right*). Arrowheads in (*B* and *C*): somatic cyst cell nuclei. (*E*) Scatter plot of RNA-sequencing data from *hs-Bam*; *bam^1/∆86^* comparing log_2_ transcript expression levels per gene for testes from flies not treated with heat shock (no HS) to testes from flies 8 h PHS. (Blue dots) *how* is one of the six genes for which transcript levels decreased by over twofold by 8 h post induction of Bam expression by heat shock in both the RNA-seq and separate microarray analysis (details in *SI Appendix*, Fig. S1). (Red dots) Transcripts from two genes increased in expression level in both the RNA-seq and microarray analysis. Diagonal lines mark twofold change. (*F*) UMAP visualization of single nuclear RNA sequencing data from the Fly Cell Atlas (5), after the nuclei in the two earliest male germ line clusters (Leiden resolution 6.0) were reclustered. (Green) nuclei scoring positive for *how*. (Magenta) nuclei scoring positive for *bam*. (White) nuclei positive for both *how* and *bam* transcripts. (*G*–*G’’*) Fluorescence images of testis apical tip showing (magenta) Bam-GFP protein and (green) *how* mRNA visualized by HCR using probes to protein coding sequences of *how*. Dashed outlines: spermatogonial cysts positive for Bam protein either (yellow) with how RNA, (white) with fewer loci of how RNA signal, or (cyan) no how RNA signal detected. [Scale bars: 25 μm in (*B*–*D*) and 12.5 μm in (*G*).]

Genetic analysis of the *Drosophila* male germ line adult stem cell lineage has revealed key players in the switch from the mitotic program of transit amplifying spermatogonia to onset of meiosis and the spermatocyte gene expression program that sets up differentiation. Germ line stem cells at the testis apical tip divide with an oriented spindle (2) so that one daughter remains next to the apical hub and maintains stem cell identity, while the daughter displaced away from the hub becomes a gonialblast, founding a clone of transit amplifying spermatogonia (Fig. 1*A*) (3). Because daughters of a single founding gonialblast divide in synchrony with incomplete cytokinesis and are enclosed by a pair of somatic cyst cells, counting the number of germ cells in each cyst can reveal the number of rounds of mitotic divisions that took place in the clone prior to the switch to the spermatocyte differentiation program. The number of transit amplifying mitotic divisions differs among *Drosophilid* species, so is under genetic control (4), with the number in wild-type *Drosophila melanogaster* almost always exactly four (Fig. 1*A*).

Mutational analysis has identified three interacting proteins—Bag of marbles (Bam), Benign gonial cell neoplasm (Bgcn), and Tumorous testes (Tut), that are required for spermatogonia to stop proliferating and become spermatocytes (6–9). While Bgcn and Tut have RNA recognition motifs (9, 10), Bam does not contain any known RNA-binding domains. Bam appears to form a bridge in a ternary complex, with sequences in the Bam N-terminal third binding Tut and sequences in the Bam C-terminal third binding Bgcn (9). Bam protein expression detected by immunofluorescence staining showed the protein present in 4-cell cysts, higher in 8-cell cysts, and remaining high during premeiotic S phase, after which the protein quickly disappeared (4). Gene dosage experiments indicated that the number of transit amplifying divisions is set by the length of time it takes Bam protein to reach a critical threshold (4). In flies with one mutant and one wild type allele of *bam*, spermatogonial cysts sometimes undergo one or occasionally more rounds of mitotic divisions before switching to spermatocyte differentiation. Conversely, in flies carrying a frameshift allele that deleted the Bam C-terminal PEST sequence, Bam protein accumulated more rapidly in transit amplifying spermatogonia and the cells often switched to spermatocyte state a round earlier, resulting in spermatocyte cysts with 8 germ cells rather than the normal 16 (4).

Bam protein has been shown to directly bind the Caf40 subunit of the CCR4-NOT deadenylation complex (CNOT9 in humans), suggesting that Bam may target RNAs for degradation or translational repression by recruiting the CCR4-NOT complex (11). While some targets of Bam in the male germ line are known, none of the genes identified so far appear to be key regulators in the switch from mitosis to meiosis downstream Bam. Previous work showed that Bgcn, Tut, and possibly Bam bind the 3′UTR of *mei-P26* and reduce expression of Mei-P26 protein. However, overexpression of *mei-P26* did not prevent the switch to spermatocytes, and decreasing *mei-P26* did not allow the switch to occur, suggesting that Mei-P26 is not the key target of Bam for the switch from mitosis to meiosis in males (9, 12).

Here, we show that a key role of Bam and Bgcn in the switch from mitosis to meiosis in the *Drosophila* male germ line is to repress expression of the RNA-binding protein Held Out Wings (How), a homolog of mammalian Quaking, with roles in alternative RNA processing, export, and translation. How protein is normally down-regulated in germ cells soon after Bam protein becomes expressed but remained high in the TA spermatogonia that overproliferate in *bam* mutant males. Strikingly, knocking down *how* expression in *bam* mutant spermatogonia by RNAi allowed the germ cells to switch to spermatocyte state and differentiate. Reciprocally, forced expression of nuclear targeted How blocked differentiation of otherwise wild-type spermatogonia, even in the presence of Bam protein. Consistent with the model that Bam down-regulates *how* RNA by recruiting Caf40 and the CCR4-NOT RNA degradation complex, knockdown of *Caf40* in early spermatogonia resulted in persistence of *how* RNA and overproliferation of TA cells, even in the presence of Bam protein. How is an RNA-binding protein that regulates alternative RNA processing, export, and translation. Our findings reveal that an irreversible cell fate transition from mitosis to terminal differentiation in an adult stem cell lineage is controlled by a regulatory cascade of RNA-binding proteins.

## Results

### Downregulation of *how* Is an Early Consequence of Bam Action.

Function of *bag-of-marbles* (*bam*) is required for proliferating spermatogonia to turn down expression of How. In testes wild-type for *bam*, immunofluorescence staining showed that How protein, present in the nucleus of early germ cells, is down-regulated in mid-stage transit amplifying spermatogonia, soon after the Bam protein was first detected by immunofluorescence staining (Fig. 1*B*), as previously documented by Monk et al. (13). Some mid-stage spermatogonia up regulating Bam protein still showed staining for How protein in their nuclei (Fig. 1*B*, yellow dotted outline), while adjacent Bam positive cysts lacked detectable How protein (Fig. 1*B*, white dotted outline). How protein continued to be detected in the nuclei of the somatic cyst cells that enclose spermatocyte cysts (Fig. 1 *B* and *C*, arrowheads), as well as in the testis sheath, but was below the level of detection in germ cells by early spermatocyte stages. In contrast, How protein persisted at high levels in the nuclei of the spermatogonia that continued to overproliferate in *bam* mutant males (Fig. 1*D*).

When *bam* mutant spermatogonia were induced to differentiate in response to a burst of Bam expression under heat shock control, downregulation of *how* RNA was one of the earliest responses detected both by RNA-Seq and independent microarray analysis of whole testes Fig. 1*E* and *SI Appendix*, Fig. S1). In the heat shock Bam time course strategy developed by Kim et al. (14), when *bam; hs-Bam* males were shifted as late pupae to 37 °C for 30 min to induce expression of Bam then returned to 25 °C, a wave of spermatogonia initiate differentiation, complete mitosis and a final S phase by 24 h post heat shock (PHS), and differentiate into spermatocytes, with onset of expression of early spermatocyte-specific transcripts beginning by 24 h PHS (*SI Appendix*, Fig. S1).

Analysis of PolyA+ RNAs expressed in whole testes at early time points in the heat shock-Bam differentiation time course showed that downregulation of *how* RNA was among the earliest changes detected. The level of *how* transcripts detected fell by >twofold by 8 h PHS, long before the germ cells began to express spermatocyte-specific markers. *how* was one of six genes showing greater than twofold decrease in transcript level by 8 h PHS by both RNA-sequencing and microarray analysis (Fig. 1*E* and *SI Appendix*, Fig. S1*A*). In the gene expression comparisons in Fig. 1*E* and *SI Appendix*, Fig. S1, only those genes that met the following criteria were colored blue (for down-regulated) or red (for upregulated): Both RNA-Seq and independent microarray analysis of testes from *hs-Bam;bam* flies subjected to heat shock showed transcript levels changed >twofold compared to the same genotype not subjected to heat shock, and testes from control *bam* mutant flies lacking the *hs-bam* did not show >twofold change expression by microarray analysis for the same gene at the indicated times PHS compared to testes from *bam* flies not subjected to heat shock (*SI Appendix*, Fig. S1). Transcripts from additional genes became down-regulated >twofold by later time points, with the number of genes with lowered transcripts growing from six at 8 h PHS to 28 (16 h), 83 (24 h), and 114 by 32 h PHS, by which time transcripts from a number of genes expressed specifically in spermatocytes began to be detected (*SI Appendix*, Fig. S1 *D* and *E*).

Reclustering of snRNA-seq data from early germ cells generated by Li et al. (5) showed that most early germ cell nuclei expressed either *how* RNA or *bam* RNA, while a small subset of the nuclei were positive for both (Fig. 1*F*, white dots, merge). Fluorescence in situ hybridization (FISH) confirmed that levels of *how* RNA became down-regulated as germ cells differentiate into spermatocytes (*SI Appendix*, Fig. S2 *B*–*E*) and abruptly decreased in early germ cells soon after onset of Bam protein expression in wild-type testes (Fig. 1*G* and *SI Appendix*, Fig. S2 *C*–*E*). In testes carrying a *Bam-GFP* transgene to allow visualization of Bam protein expression in mid to late transit amplifying spermatogonia, Hybridization Chain Reaction (HCR) FISH with probes recognizing the How protein coding sequence (*SI Appendix*, Table S1) detected *how* transcripts in the nucleus and cytoplasm of cells near the testis tip, apical to the region where germ cells expressing Bam-GFP were detected (Fig. 1*G* and *SI Appendix*, Fig. S2 *C*–*E*). *how* in situ signal was apparent in the cytoplasm of germ cells in some early spermatogonial cysts expressing Bam-GFP (yellow outline), while other Bam positive cysts showed little or no signal for *how* RNA (white and cyan outlines, respectively) (Fig. 1*G* and *SI Appendix*, Fig. S2 *C*–*E*), suggesting that the level of *how* RNA in spermatogonia decreases rapidly once Bam protein becomes expressed. HCR FISH with a different probe set, directed against the 3’UTR associated with *how* mRNAs that encode nuclear targeted How(L) protein isoforms (*SI Appendix*, Table S2) also detected *how* RNA signal at the testis apical tip that decreased with distance from the testis hub, like signal from the *how* protein coding region probe correlating with progression of transit amplifying spermatogonia to more mature stages (*SI Appendix*, Fig. S2*B*).

### *how* Is a Key Target of Bam for the Proliferation to Differentiation Switch.

**Fig. 2.**
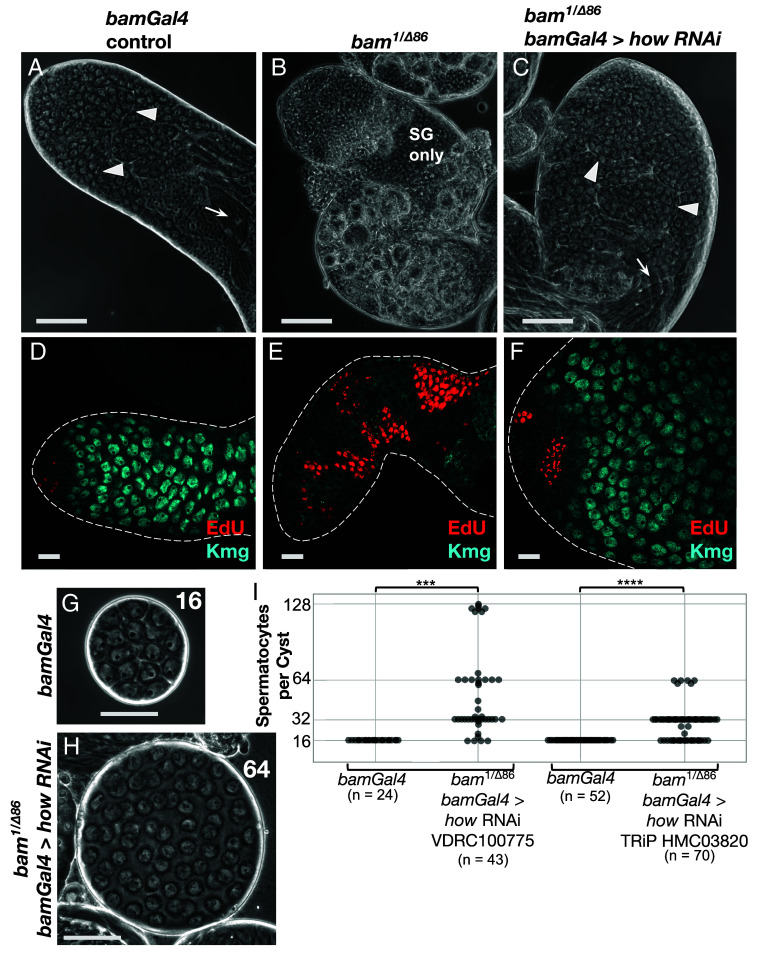
Knocking down *how* allowed spermatogonia lacking *bam* to differentiate into spermatocytes. (*A*–*C*) Phase contrast images of apical regions of testis in live squash preparations. (*A*) *bamGal4* expression driver only. (*B*) *bam^1/∆86^*. (*C*) *bam^1/∆86^*; *bamGal*4 > *how* RNAi (VDRC100775). All flies were raised under the same temperature shift regimen used for RNAi. Arrowheads: spermatocytes. Arrows: spermatid bundles. SG: spermatogonia. (Scale bar: 100 μm.) (*D*–*F*) Immunofluorescence images of testis apical tips stained for (red) EdU to mark nuclei in S phase and (blue) Kmg to mark spermatocyte nuclei. (*D*) *bamGal4* driver only control. (*E*) *bam^1/∆86^*. (*F*) *bam^1/∆86^*; *bamGal4* > *how* RNA. (Scale bar: 25 μm.) (*G* and *H*) Phase contrast images of intact spermatocyte cysts marked with the number of spermatocytes in the cyst. (*G*) *bamGal4* driver only. (*H*) *bam^1/∆86^*; *bamGal4* > *how* RNAi. (Scale bar: 50 μm.) (*I*) Number of spermatocytes per cyst from: (*Left* side) *bamGal4* sibling controls (N = 24 cysts) or *bam^1/∆86^*; *bamGal4* > *how RNAi* VDRC100775 (N = 43 cysts). (***) *P*-value = 1.716e-10, based on a Wilcoxon rank-sum test with continuity correction. (*Right* side) *bamGal4* control (N = 52 cysts) or *bam^1/∆86^*; *bamGal4* > *how RNAi* TRiP HMC03820 from Bloomington Stock #55665 (N = 70 cysts). (****) *P*-value = 1.033e-14 based on a Wilcoxon rank-sum test with continuity correction.

Knocking down expression of *how* in *bam* mutant TA spermatogonia by RNAi under control of *bam-Gal4* allowed *bam* mutant spermatogonia to differentiate into spermatocytes. Whereas squashed preparations of control testes wild-type for *bam* viewed by phase contrast microscopy showed abundant large spermatocytes and elongating spermatid bundles (Fig. 2*A*), testes from males mutant for *bam* were considerably smaller, lacked spermatocytes and spermatids, and contained large numbers of small germ cells that proliferate in cysts then eventually die (Fig. 2*B*). However, if the *bam* mutant flies also carried a *UAS-how-RNAi* construct forcibly expressed in transit amplifying spermatogonia under control of *bamGal4*, the *bam*^−/−^ germ cells successfully differentiated into spermatocytes and elongating spermatids (Fig. 2*C* and *SI Appendix*, Fig. S3), indicating that the main requirement for Bam for the switch from mitosis to meiosis in males is reducing function of How. Similar rescue allowing differentiation of *bam* mutant germ cells to spermatocytes and elongating spermatids resulted from knockdown of expression of *how* by RNAi using a nonoverlapping shRNA construct from the TRiP collection (HMC03820) (*SI Appendix*, Fig. S3 *A*–*F*). The *bam* mutant males in which *how* had been knocked down by *bamGal4* induced RNAi were fertile and produced viable offspring, similar to control males, although in both cases the number of progeny was quite low, possibly due to culturing the males at 29 °C (*SI Appendix*, Fig. S3*J*).

Testes from males mutant for *bgcn*, a binding partner of *bam*, had the same phenotype as *bam* mutants, overproliferation of transit amplifying spermatogonia that maintained expression of nuclear How protein (*SI Appendix*, Fig. S4*A*), and eventual germ cell death, with no cells at later stages of development (*SI Appendix*, Fig. S4*B*). Knockdown of *how* function in mid-to-late transit amplifying spermatogonia by RNAi under control of *bamGal4* also restored ability of *bgcn* mutant spermatogonia to differentiate into spermatocytes and elongating spermatids (*SI Appendix*, Fig. S4*C*).

Brief incubation of testes in EdU to label cells in S phase and immunofluorescence staining for the spermatocyte marker Kmg confirmed that knockdown of *how* by RNAi in mid-to-late spermatogonia under control of *bamGal4* restored the ability of *bam* mutant spermatogonia to switch to the spermatocyte state. Testes from control or RNAi knockdown flies were incubated in EdU for 5 min to label nuclei undergoing DNA replication, then immediately fixed and processed for immunofluorescence staining and imaging. Control testes from flies carrying the *bamGal4* transgene and raised under the same temperature regimen used for RNAi knockdowns showed a few small clusters of EdU-positive nuclei located near the testis apical tip, marking cysts of spermatogonia undergoing S phase in synchrony. As expected, no EdU positive nuclei were identified further from the tip, where germ cells showed expression of the spermatocyte specific marker Kmg (Fig. 2*D*). In contrast, testes from flies mutant for *bam* raised under the RNAi knockdown temperature shift regime showed many more EdU-positive cysts per testis, several with more than 16 EdU-positive nuclei, indicating spermatogonial overproliferation, and no spermatocytes expressing Kmg (Fig. 2*E*). Testes from *bam* mutant males in which expression of *how* was knocked down in late spermatogonia and early spermatocytes by RNAi under control of *bamGal4* showed many fewer EdU-positive cysts per testis, again confined to near the testis apical tip, and abundant Kmg-positive spermatocytes (Fig. 2*F*). Similar results were observed when expression of How was knocked down by a different RNAi construct expressed under control of *bamGal4* (*SI Appendix*, Fig. S3 *D*–*F*).

Notably, testes from *bam*^−/−^; *bamGal4;UAS-How RNAi* males raised under knockdown conditions were unusually wide, with many early germ cells (Fig. 2 *C* and *F* and *SI Appendix*, Fig. S3*I*). The increased width of *bam*^−/−^; *bamGal4;UAS-How RNAi* testes was likely because downregulation of *how* due to RNAi under control of *bamGal4* may occur later than in 4 to 8 cell cysts as in wild type, because of the time it takes for the *bamGal4* to be expressed, activate transcription of the RNAi construct, then for the RNAi to act upon *how* transcripts. Such delay would allow the *bam* mutant spermatogonia to go through additional rounds of mitotic transit amplifying divisions before downregulation of How. Consistent with this, spilling out individual cysts revealed that control testes raised under the knockdown temperature regimen almost always had 16 spermatocytes per cyst. In contrast, *bam^−/−^*; *bamGal4;UAS-How RNAi* testes normally had 32, 64, and sometimes more spermatocytes per cyst, indicating five, six, or more rounds of transit amplifying divisions prior to the switch to spermatocyte state, rather than the normal four (Fig. 2 *G*–*I*).

Conversely, forced expression of nuclear-targeted How but not cytoplasmic How in mid-stage spermatogonia was sufficient to largely block differentiation of otherwise wild-type spermatogonia into spermatocytes. The *how* locus encodes several transcript and protein isoforms (*SI Appendix*, Fig. S2*A*) (15). Some transcripts encode long forms of How protein that have a C-terminal nuclear localization signal [How(L)]. Others encode shorter forms of the protein that lack the nuclear localization signal [How(S)] and are cytoplasmic.

**Fig. 3.**
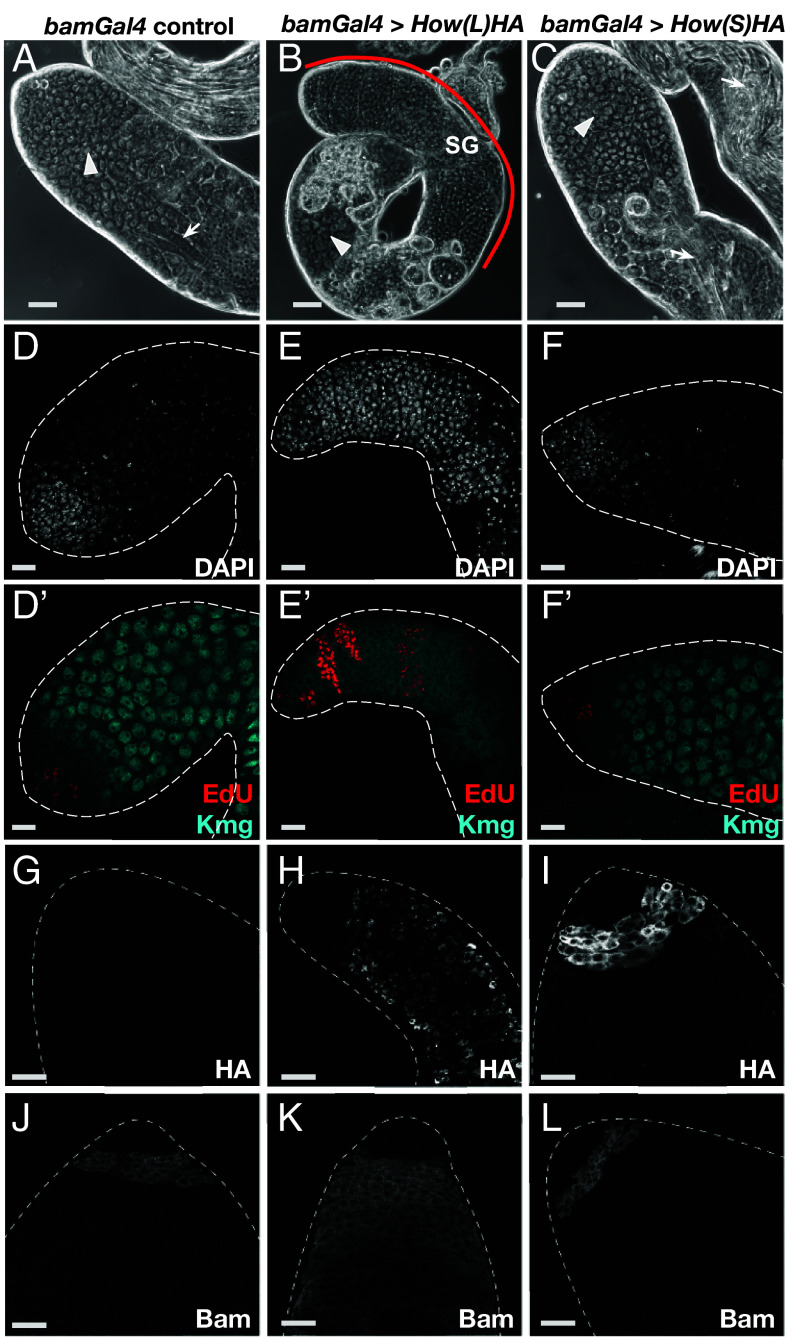
Overexpression of How(L) but not How(S) blocked the transition from spermatogonia to spermatocytes. (*A*–*C*) Phase contrast images of testis apical tips from (*A*) *bamGal4* driver only. (*B*) *bamGal4* > *UAS-How(L)HA-SV40*. (*C*) *bamGal4* > *UAS-How(S)HA-SV40*. SG: spermatogonia. Arrowheads: spermatocytes. Arrows: elongating spermatids. The red line in (*B*) marks region with overproliferating spermatogonia. (Scale bar: 50 μm.) (*D*–*F'*) Immunofluorescence images of apical tips of testes stained with (*D–F*) DAPI to mark nuclei and (*D'–F'*) EdU to mark nuclei in S phase and anti-Kmg to mark spermatocyte nuclei. (*D*) *bamGal4* driver only. (*E*) *bamGal4* > *UAS-How(L)HA-SV40*. (*F*) *bamGal4* > *UAS-How(S)HA-SV40*. (Scale bar: 25 μm.) (*G*–*I*) Immunofluorescence images of testis apical tips stained with anti-HA to detect the forcibly expressed How(L) and How(S). (*G*) *bamGal4* driver alone control. (*H*) *bamGal4*, *UAS-How(L)HA-SV40* showing nuclear localization of How(L). Note persistence of How(L)HA expression in the overproliferating spermatogonia. (*I*) *bamGal4* > *UAS-How(S)HA-SV40* showing cytoplasmic localization of How(S). Note shut off of How(S)HA expression in differentiating spermatocytes. (*J*–*L*) Immunofluorescence images of testis apical tips stained with anti-Bam. (*J*) *bamGal4* control. (*K*) *bamGal4* > *UAS-How(L)HA-SV40*. (*L*) *bamGal4* > *UAS-How(S)HA-SV40*. (Scale bar: 25 μm.)

Flies bearing a transgene with UAS sequences controlling inducible expression of How(L) protein (isoform A in *SI Appendix*, Fig. S2*A*) cloned in-frame with a C-terminal HA epitope tag followed by terminator sequences from SV40 [*UAS-How(L)HA-SV40*] were crossed to flies bearing *bamGal4* to drive expression in mid-to-late transit amplifying spermatogonia. The mated flies were cultured at 25 °C for 3 d then adults were removed and the progeny shifted to and maintained at 29 °C to boost expression from the *UAS-How(L)HA-SV40* transgene. Under these conditions, testes from the newly eclosed *bamGal4; UAS-How(L)-HA-SV4* progeny had extensive apical regions filled with small germ cells (Fig. 3*B* red bracket; compare to Fig. 3*A*), followed by areas with dying cells, as in *bam* mutant testes. Some testes also had a few cysts containing spermatocytes or postmeiotic spermatids, usually located far down the testis away from the apical tip, distal to the region with cell death.

Immunofluorescence staining after a short pulse of EdU revealed that most (~80%) of the *bamGal4; UAS-How(L)HA SV40* testes had a much larger than normal number of cysts with germ cells undergoing synchronous DNA replication per testis than controls (Fig. 3*E’*). The germ cell cysts subjected to forced expression of How(L) often had more than 16 nuclei undergoing S phase, indicating overproliferation. In addition, the nuclei remained small and stained brightly with DAPI, unlike in spermatocytes, and lacked expression of the spermatocyte marker Kmg (Fig. 3*E’*). Immunofluorescence staining with anti-HA confirmed that How(L)-HA was localized to germ cell nuclei and that the *bamGal4* driver did not force expression of *How(L)HA* in germ line stem cells and early spermatogonia (Fig. 3*H*). Notably, immunofluorescence staining with anti-Bam showed that Bam protein was expressed in the spermatogonia that overproliferated when *How(L)HA-SV40* was forcibly expressed, indicating that the failure of spermatogonia to differentiate was not due to repression of Bam by nuclear targeted How(L) (Fig. 3*K*).

In contrast, testes from males expressing *UAS-How(S)HA-SV40* (isoform B in *SI Appendix*, Fig. S2*A*, which lacks the NLS) under control of *bamGal4* raised under the same conditions did not show massive overproliferation of spermatogonia, but instead had a modest population of small germ cells at the testis apical tip followed by plentiful differentiating spermatocytes, visible as large cells with large nuclei in unfixed squashed preparations viewed by phase contrast microscopy (Fig. 3*C*). Immunofluorescence staining confirmed that the switch to spermatocyte state occurred after a limited number of mitotic divisions when *How(S)HA* was forcibly expressed in mid-to-late spermatogonia. Testes from *bamGal4; UAS-How(S)HA-SV40* males subjected to a brief incubation in EdU showed only a small number of EDU positive cysts, all of which were close to the apical tip. The testes also had many spermatocytes, marked by large nuclei positive for Kmg protein, starting from within a few cell diameters of the testis apical tip (Fig. 3*F’*). Immunofluorescence staining with anti-HA confirmed that How(S)HA was cytoplasmic (Fig. 3*I*). As expected for the *bamGal4* driver, anti-HA was not detected in very early germ cells at the testis tip. Together these data indicate that it is nuclear rather than cytoplasmic forms of How protein that block the ability of spermatogonia to differentiate into spermatocytes.

**Fig. 4.**
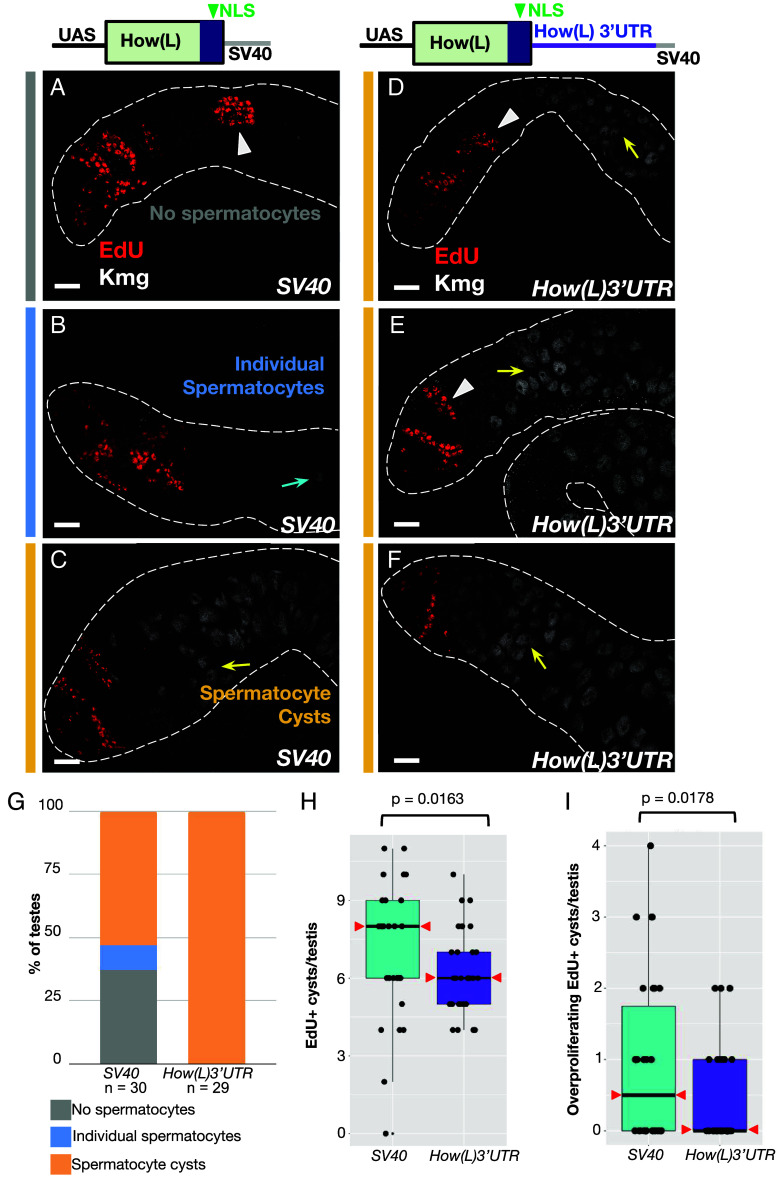
Adding the How(L) 3’UTR reduced the effect of forced expression. (*A*–*F*) *Top*: cartoons depicting design of the overexpression constructs. (*Left*) *UAS-How(L)HA* with the SV40 terminator, (*Right*) *UAS-How(L)HA* with the 2424nt 3’UTR from How(L) (*SI Appendix*, Fig. S2*A*) added before the SV40 terminator. Immunofluorescence images of testes stained for (white) Kmg to mark spermatocyte nuclei and (red) EdU to label cysts in S phase in the phenotypic classes indicated in (*G*). (*A*–*C*) *bamGal4* > *UAS-How(L)HA-SV40*. (*D*–*F*) *bamGal4* > *UAS-How(L)HA-3’UTR-SV40*. Flies were grown at 18 °C for their entire life. Arrowheads: EdU positive cysts. Blue arrow: single Kmg positive spermatocyte. Yellow arrows: spermatocytes in cysts. [Scale bar in (*A*–*F*): 25 μm.) (*G*) Graph of the percentage of testes from each genotype with either (gray) no spermatocytes detected, (blue) single spermatocytes or (orange) cysts of multiple spermatocytes, scored based on the spermatocyte marker Kmg. *SV40* n = 30 testes. *How(L)3’UTR* n = 29 testes. Percentages were compared using a two-sample *t* test of proportions. Percentage of testes containing spermatocyte cysts (*P* = 2.5 × 10^−5^) and percentage of testes containing any spermatocytes (*P* = 3.0 × 10^−4^) differed significantly between the two genotypes. (*H*) Distribution of the number of EdU positive cysts in *bamGal4* > *UAS-How(L)-SV40* testes and *bamGal4* > *UAS-How(L)-3’UTR-SV40* testes. Statistical tests performed via one-way between-groups ANOVA. (*I*) Number of overproliferating (>16 nuclei) EdU positive cysts from testes overexpressing *How(L)-SV40* (n = 30 testes) compared to *How(L)-3’UTR-SV40* (n = 29 testes). *P*-value calculated as for (*H*). The median in *How(L)-3’UTR-SV40* was zero overproliferating cysts/testis. Medians marked by red arrowheads in all conditions.

Addition of 3’UTR sequences from *how(L)* mRNA reduced the effect of forced expression of *How(L)HA* on the ability of spermatogonia to stop proliferating and differentiate into spermatocytes. *How(L)* mRNA isoforms have overlapping 1566nt - 2424nt 3’UTRs, which do not share sequences with the much shorter 3’UTRs of mRNA isoforms that encode How(S) proteins (15) (*SI Appendix*, Fig. S2*A*). The 2424nt How(L) 3’UTR from *how* mRNA isoform A (*SI Appendix*, Fig. S2*A*) was cloned into the *UAS-How(L)HA-SV40* overexpression construct between the HA tag and the SV40 terminator to generate *UAS-How(L)HA-How(L)3’UTR-SV40* (Fig. 4, *Top* diagram) and introduced into flies as a transgene (*Methods*).

Flies bearing either the newly constructed *UAS-How(L)HA-How(L)3’UTR-SV40* transgene [hereafter termed *UAS-How(L)-3’UTR*] or a parallel *UAS-How(L)-SV40* transgene lacking the *How(L) 3’UTR* [hereafter termed *UAS-How(L)-SV40*] inserted at the same genomic site were then crossed to flies bearing *bamGal4* to drive expression starting in mid-to-late spermatogonia and the progeny grown continuously at 18 °C. Under these conditions, forced expression of *UAS-How(L)-SV40* had a range of phenotypes, visualized, and scored by EdU labeling and anti-Kmg staining. Some testes (37%) had no spermatocytes detected at all (Fig. 4*A*), while the remaining 63% of testes contained at least some individual Kmg-positive spermatocytes (Fig. 4*B*), with 53% of the testes scored containing entire cysts of Kmg-positive spermatocytes (n = 30 testes) (Fig. 4 *C* and *G*). However, in flies in which the *UAS-How(L)-3’UTR* construct was forcibly expressed under control of *bamGal4* at 18 °C, 100% of testes had at least some spermatocyte cysts (n = 29 testes) (Fig. 4 *D*–*G*). Briefly incubating testes in EdU to label nuclei in S phase revealed that flies overexpressing How(L)-HA with and without the How(L)3’UTR both contained spermatogonial cysts that had undergone additional rounds of proliferation beyond the normal 4, visualized as EdU positive cysts with many more than 16 labeled nuclei (Fig. 4 *A*–*F* and *H*). However, *UAS-How(L)-3’UTR* testes had overall less spermatogonial overproliferation, based on fewer cysts undergoing S phase per testis (Fig. 4*H*) and fewer cysts with more than 16 EdU labeled nuclei per cyst (Fig. 4*I*), compared to testes from flies overexpressing *UAS-How(L)-SV40* [without the How(L) 3’UTR] grown in parallel. The milder effect of forced overexpression of *How(L)-3’UTR* compared to *How(L)-SV40* under control of *bamGal4* raised the possibility that Bam may downregulate How expression via the How(L) 3’UTR.

### Bam May Downregulate *how* by Recruiting the CCR4-NOT Complex.

**Fig. 5.**
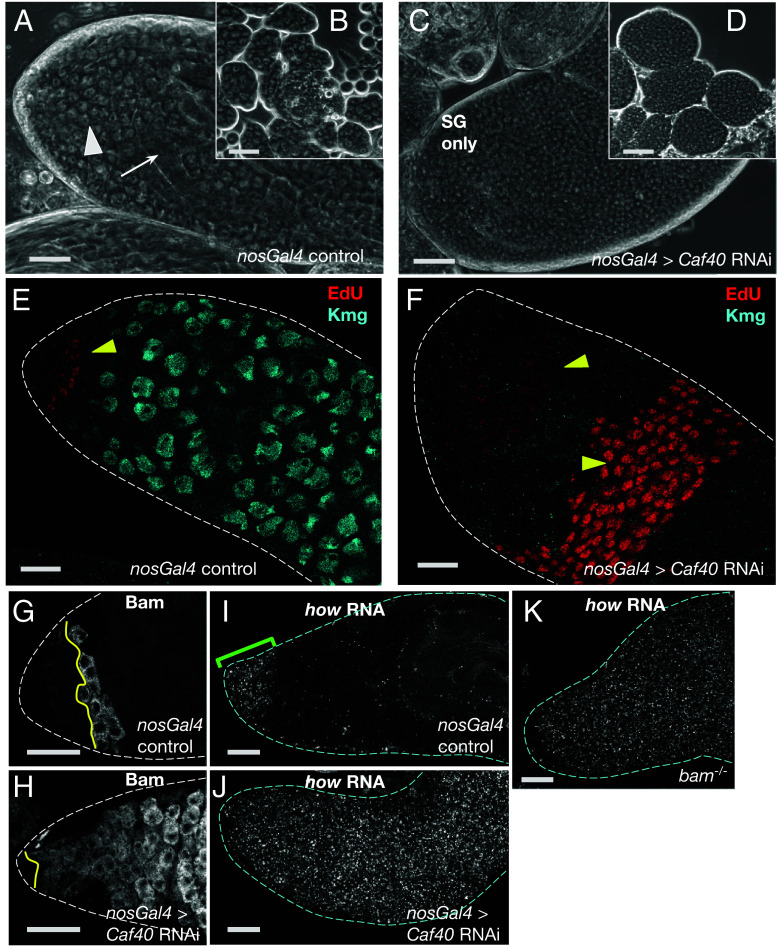
The CCR4-NOT component Caf40 is required for *how* repression. (*A*–*D*) Phase contrast images of testis apical tips from males carrying (*A* and *B*) *nosGal4* driver only control and (*C* and *D*) *nosGal4* > *Caf40 RNAi* (TRiP HMS05850 Bloomington stock #67987). White arrowhead: spermatocytes. White arrow: spermatid bundles. SG = spermatogonia. (*B* and *D*) Spilled out cysts from (*B*) *nosGal4* control testes including many spermatocyte cysts as well as more uncommon cysts of smaller spermatogonia. Spilled out cysts from (*D*) *nosGal4* > *Caf40* RNAi males contained many small cells and no spermatocytes. (*E* and *F*) Immunofluorescence images of testes apical tips from (*E*) *nosGal4* driver only control and (*F*) *nosGal4* > *Caf40* RNAi labeled with (red) EdU to mark nuclei in S phase and (cyan) anti-Kmg to mark spermatocyte nuclei. Yellow arrowheads: cysts of EdU positive spermatogonia. (*G* and *H*) Immunofluorescence images of testis apical tips stained with anti-Bam from (*G*) *nosGal4* and (*H*) *nosGal4* > *Caf40* RNAi flies. Yellow line: boundary between early spermatogonia and Bam positive spermatogonia. (*I*–*K*) Apical tips of testes stained for *how* RNA by HCR with probes complementary to the How protein coding sequence. (*I*) *nosGal4* control. Green bracket: region with *how* transcripts in spermatogonia. (*J*) *nosGal4* > *Caf40* RNAi. (*K*) *bam^1/∆86^*. [Scale bar: 50 μm (*A*–*D*); 25 μm (*E*–*K*).] All control flies were raised under the same temperature regimen used for the RNAi (3 d at 25 °C, then cultured at 29 °C).

The reduction of *how* transcript levels in transit amplifying spermatogonia soon after the appearance of Bam protein in the cytoplasm (Fig. 1*G* and *SI Appendix*, Fig. S2 *C*–*E*) suggested that Bam may downregulate *how* at least in part due to effects on the *how* RNA. Protein structure studies by Sgromo et al. showed that a 23 amino acid sequence near the N terminus of the Bam protein binds in a groove of Caf40, a subunit of the CCR4-NOT complex (11). Further, Sgromo et al. showed that when Bam or an N-terminal fragment of Bam containing the Caf40-binding motif (CBM) was tethered to a luciferase reporter RNA, it was able to recruit CCR4-NOT for transcript degradation, decreasing levels of luciferase RNA and protein (11). These findings raise the possibility that Bam and Bgcn may downregulate *how* by recruiting Caf40. Consistent with this, knockdown of *Caf40* in early spermatogonia by RNAi under control of *nosGal4* resulted in massive overproliferation of small germ cells, similar to the phenotype observed in *bam* mutant males (Fig. 5*C* and *SI Appendix*, Fig. S5 *B*–*E*). Digesting the testis sheath to spill out intact cysts confirmed that the overproliferating small cells were organized in cysts (Fig. 5*D*), as in *bam* mutants. Brief incubation in EdU confirmed that large clusters of germ cells were undergoing DNA synthesis in synchrony in testes in which expression of *Caf40* had been knocked down under control of *nosGal4*, while immunofluorescence staining confirmed failure to turn on expression of the spermatocyte marker Kmg (Fig. 5*F*), as in *bam* mutant males. Strikingly, immunofluorescence staining with anti-Bam revealed that the hundreds of small germ cells that overproliferated when *Caf40* was knocked down by RNAi under control of *nosGal4* expressed Bam protein (Fig. 5*H*), indicating that they were germ cells and that the failure to differentiate was not due to failure to express Bam.

Analysis by fluorescence HCR in situ hybridization (FISH) confirmed that *how* transcripts remained in the germ cells that overproliferated after function of the CCR4-NOT component *Caf40* was knocked down by RNAi. In testes from control males carrying the *nosGal4* driver but not the *UAS-Caf40 RNAi* construct (Fig. 5*I*) raised under RNAi temperature shift conditions, *how* mRNA was detected in early germ cells near the testis apical tip but was not detected in the spermatocyte region further down the testes (as in Fig. 1). However, despite the expression of Bam protein, *how* transcripts remained high in the overproliferating germ cells that accumulated in testes in which *Caf40* had been knocked down in early germ cells under control of *nosGal4*, as observed in *bam^−/^*^−^ males (Fig. 5 *J* and *K*). The persistence of *how* transcripts despite the presence of Bam protein suggested that Caf40 does not act upstream of Bam but instead might be part of the mechanism through which Bam protein down-regulates *how* RNA.

## Discussion

### A Key Function of Bam in Spermatogonia Is Repression of *how*.

**Fig. 6.**
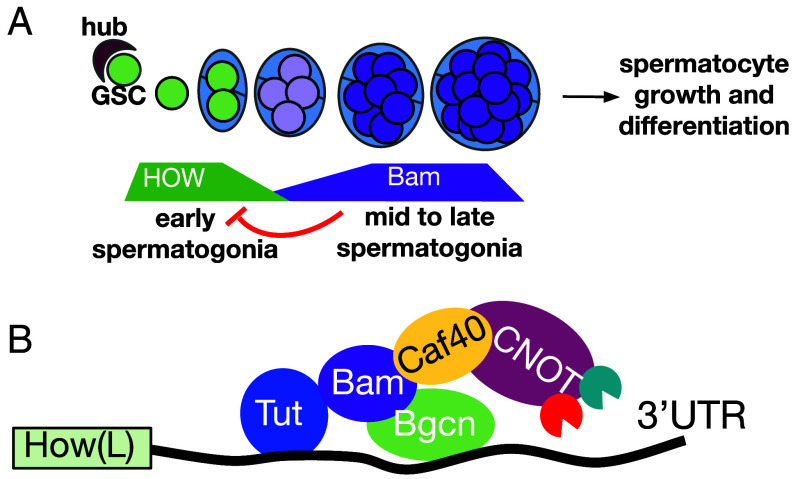
Model for mechanism of Bam action on *how* RNA. (*A*) Cartoon of *How* (green) and *Bam* (purple) protein expression in the germ line stem cell and spermatogonia. Levels of *how* decrease as Bam expression appears at the four cell cyst stage. Bam levels increase in mid-to-late spermatogonia until sufficient to repress expression of *How*, allowing the subsequent switch from proliferation to differentiation. (*B*) Model for regulation by Bam, Bgcn, and Tut: Bam protein binds the CCR4-NOT subunit Caf40, acting as an adaptor between the CCR4-NOT complex and its target transcripts.

Our results indicate that the major role of Bam and Bgcn in the switch from mitotic proliferation to onset of the meiotic program in the male germ line is to down-regulate expression of How, the *Drosophila* homolog of mammalian Quaking (Fig. 6*A*). Most telling, male germ cells lacking *bam* function can differentiate if expression of *how* is knocked down in mid-to-late stage transit amplifying spermatogonia by RNAi. In addition, forced expression of a nuclear targeted isoform of How, How(L), was sufficient to cause spermatogonia to overproliferate and largely fail to become spermatocytes. Strikingly, spermatogonia subjected to forced expression of How(L) expressed abundant Bam protein, indicating that the failure to differentiate was not due to How(L) repressing expression of Bam.

Our finding that knockdown of *Caf40* by RNAi driven by *nosGal4* led to overproliferation of spermatogonial cysts and persistence of *how* RNA and protein, even though Bam protein was present, suggested that *Caf40* is required for Bam to downregulate *how*. Sgromo et al. showed that Bam protein has a 23-amino acid N-terminal domain that binds in a groove of *Caf40* (homolog of human NOT9), a subunit of the CCR4-NOT complex (11). Through this Caf40-binding motif (CBM) domain, Bam tethered to a reporter RNA was able to recruit the CCR4-NOT complex to degrade the reporter RNA (11). Bam protein appears to participate in a ternary complex, bridging between the RNA-binding protein Tut bound to sequences in the Bam N-terminal third and the RNA-binding protein Bgcn bound to sequences in the Bam C-terminal third (9). Consistent with models suggested by others, we propose that Bam, recruited to target RNAs by its structural partners Bgcn and Tut, may act as an adaptor to recruit the CCR4-NOT deadenylation complex to destabilize the bound RNA and/or lead to its translational repression (9, 11, 16) (Fig. 6*B*). In support of the model that Bam targets *how* RNA for degradation and/or translational repression by recruiting CCR4-NOT, knockdown of several CCR4-NOT subunits by RNAi under control of the *bamGal4* driver showed a mild overproliferation phenotype in spermatogonial cysts (16).

Bam and Bgcn have been shown to downregulate expression of E-Cadherin via sequences in the E-Cadherin 3’UTR (17). Assays conducted in S2 cells carrying reporter constructs with the Firefly *luciferase* coding sequence attached to the 3’UTR from E-Cadherin showed that expression of luciferase was down-regulated compared to a renilla control with a heterologous 3’UTR if Bam and Bgcn were coexpressed in the cells. The inhibition of luciferase expression was not observed when either Bam or Bgcn was expressed without its partner, but was detected if Bam was tethered to the E-Cadherin 3’UTR. Bgcn, Bam, and Tut have also been shown to repress expression of Mei-P26 via sequences in the *mei-P26* 3’UTR (9, 12).

Bam serves as a differentiation factor in both the male and female germ lines (7, 8). The regulatory logic is similar: In both cases, action of *bam* down-regulates a factor required for maintenance of the precursor state. In *Drosophila* females, the germ line stem cell state is maintained by *nanos* and *pumilio*, which are thought to repress translation of transcripts that drive differentiation (8, 18–20). Loss of function of *nanos* or *pumilio* in females resulted in loss of germ line stem cells to differentiation (19). In female early germ cells, expression of *nanos* protein was abruptly down-regulated when Bam protein became expressed in late cystoblasts. Nanos protein expression persisted in the oogonia that overproliferate in *bam* mutant ovaries, and the downregulation of *nanos* in response to Bam depended on sequences in the *nanos* 3’UTR (8, 21). Similarly, in males, How protein is down-regulated when Bam becomes expressed in mid stage transit amplifying spermatogonia, How protein persisted in spermatogonia mutant for *bam*, and addition of the How(L) 3’UTR somewhat reduced the effects of forced expression of How(L).

The timing of Bam expression and action is different in male than in female germ cells. In females, Bam protein becomes upregulated enough to be detected by immunofluorescence staining in the late cystoblast, the immediate daughter of a stem cell division, which will found a clone of female germ cells that will eventually generate one oocyte and fifteen nurse cells. In males, Bam protein is upregulated later, during the spermatogonial transit amplifying divisions, so that it was initially detected by immunofluorescence staining in four cell cysts, appeared to increase in level by the 8 cell stage, and remained present through premeiotic S phase before being abruptly degraded (4). In both sexes, expression of How protein in germ cells appears reciprocal to expression of Bam. In females, How protein was present in the nucleus of female germ line stem cells but was abruptly down-regulated by the two-cell stage (22). Male germ line stem cells also showed nuclear How, which persisted in gonialblasts and two cell transit amplifying stages but dropped precipitously once Bam protein was expressed and began to accumulate (13, this study).

In males, knockdown of *how* in germ line stem cells and early transit amplifying cells under control of *nosGal4* led to loss of germ line stem cells, as did induction of germ line clones homozygous for a strong loss of function *how* allele (13). Germ cells homozygous mutant for *how* appeared stalled in G2 at the two cell cyst stage, likely due to defects in expression of *Cyclin B*, and eventually died (13). The effect of loss of How function in female germ line stem cells was different: Induction of germ line clones mutant for *how* led to gradual loss of female germ line stem cells, possibly to differentiation, but not to cell cycle arrest and apoptosis (22). These results suggest that although the regulatory relationship between How and Bam may be similar, the roles of How (and Bam) in early germ cells in the two sexes likely differs.

Monk et al. (13) observed that using *nosGal4* to drive overexpression of *How(L)-SV40* in early germ cells (approximately the time when How is normally expressed) caused shortening of cell cycle time in TA spermatogonia, likely due to increased expression of Cyclin B, and frequent 32 cell spermatocyte cysts, indicating switching from spermatogonia to spermatocytes after 5 rather than the usual 4 rounds of transit amplifying mitotic divisions. Monk et al. proposed that How(L) represses expression of Bam protein in early spermatogonia (13). In contrast, we found that using *bamGal4* to drive forced expression of *How(L)-SV40* later, a time when it is normally down-regulated, resulted in massive overproliferation of spermatogonia. Strikingly, Bam protein was expressed in the spermatogonia overproliferating under these conditions, arguing that How(L) does not repress expression of Bam in later stage spermatogonia.

Our data suggest that Bam down-regulates expression of How in mid-to-late transit amplifying spermatogonia, possibly through the How(L) 3’UTR, which was not included in the *How(L)-SV40* construct. Such a mutual repression relationship may provide a mechanism to convert a gradual rise in Bam expression (perhaps driven at the level of transcription) into a sharp switch in cell state. In early transit amplifying male germ cells, abundant How(L) protein may repress premature accumulation of Bam protein and maintain a stem cell competent, early TA state. As expression *of bam* transcript and protein rise, however, Bam may reach levels sufficient to bind via a Tut-Bam-Bgcn complex to all or most of the How(L) mRNA molecules, targeting them for translational repression and/or degradation and so throwing the switch to onset of differentiation. Alternatively, the 32 spermatocyte cell cysts detected by Monk et al. could be due to the observed shortening of the cell cycle in spermatogonia after forced expression of How(L) under control of *nosGal4* (13) rather than to How(L) repression of *bam* in early spermatogonia. As shown by Insco et al. shortening the cell cycle can allow more cysts to complete 5 rounds of cell division before Bam protein reaches the critical level required for the switch to spermatocyte state (4).

The fact that testes from *bamGal4>UAS-How(L)-3'UTR* males still showed some aberrant overproliferation of spermatogonia may be because getting the proportions of *how* RNA and Bam protein just right so that all the *how* RNA can be bound and repressed/degraded is not easy. The amount of How(L)-3'UTR mRNA produced in the *bamGal4>UAS-How(L)-3'UTR* males may titrate out the endogenous Bam protein and so overwhelm the capacity of Bam to repress either the *UAS-How(L)* or endogenous *how* RNA.

A central lesson from genetic analysis of the *Drosophila* male and female germ line adult stem cell lineages is that the switch from precursor cell state to onset of differentiation is controlled by a cascade of RNA-binding proteins. In each case, Bam protein with its RNA-binding partner Bgcn acts to trigger differentiation by downregulation of another RNA-binding protein. In females, the key target is the translational inhibitor *nanos* (8). Here, we show that in males the key target is the RNA-binding protein How. It will be interesting in future work to test whether mRNAs bound and regulated by How(L) in early spermatogonia maintain the germ cells in a proliferative state or block their differentiation. Our results suggest that it will also be important to investigate whether similar regulation by RNA-binding proteins controls the switch from proliferation to differentiation in adult stem cell lineages in other organisms.

## Methods

### Fly Strains and Husbandry.

Flies were raised on molasses food. Overexpression and knockdown crosses were raised at 25 °C for 3 d, then shifted to 29 °C after discarding adults, unless otherwise noted. RNAi lines were obtained from The Vienna Drosophila Resource Center (VDRC): UAS *how* RNAi (100775), UAS *caf40* RNAi (101462), and the Bloomington Drosophila Stock Center: UAS *how* RNAi (Bloomington stock #55665, containing TRiP line HMC03820) and UAS *caf40* RNAi (67987, containing TRiP line HMS05850). Results using the How RNAi line VDRC100775 are shown throughout except for Fig. 2*I* and *SI Appendix*, Figs. S3 and S6, which also show results from knocking down How using the TRiP RNA construct HMC03820 carried in Bloomington stock #55665. For Fig. 4, flies were raised at 18 °C throughout. For the heat shock time course, flies were raised at 25 °C until there were mid- and late-stage pupa. Then bottles were placed in a 37 °C water bath for 30 min to induce heat shock and returned to 25 °C, as spelled out in ref. 14. *UAS-How(Long)-3xHA* and *UAS-How(Short)-3xHA* fly stocks with an SV40 heterologous 3'UTR were a gift from T. Volk, Weizmann Institute of Science, Rehovot, Israel (23). Two Gal4 driver lines were used: *nanosGal4*-*VP16* for germ line stem cells and early spermatogonia and *bamGal4* for the mid- to late spermatogonia (24, 25). Gal4 driver strains for knockdowns also contained *UAS-Dicer2*. The *bam* alleles were *bamP*-*bam-GFP* (25), *bam^1^* (6), and *bam*^Δ86^ (26).

### Phase Contrast Microscopy.

For testis squashes, testes from 0 to 1 d old males were dissected in PBS. Whole testes or cysts were flattened into a monolayer under a coverslip by wicking away PBS and observed by phase contrast microscopy using a Zeiss Axioskop. Images were taken with a Spot Imaging camera and software. To count the number of spermatocytes per cyst, dissected testes were treated with 0.5 mg/mL collagenase (Sigma C7657) + 0.5 mg/mL dispase (Worthington, LSO2109) in PBS on a slide for 1.5 to 3 min (larger testes burst open sooner) (as described in ref. 27). The reaction mix was replaced with PBS and cysts were gently flattened under a coverslip by wicking away liquid before counting and imaging.

### Transgenics.

Plasmids containing *how* coding sequences with HA tags expressed under control of UAS were a gift from Talila Volk (23). The *how*(L) coding sequence (FBgn0264491, isoform A on FlyBase) was amplified and inserted into a pUASTattB vector at NotI and XhoI restriction sites (15). To add the *how*(L) 3’UTR (FBtr0084177) was amplified in two parts from *bam* testis cDNA (to exclude the intron) and inserted upstream of the SV40 polyadenylation site, using XhoI and XbaI restriction sites. Differences in the *how* coding sequence from published fly genome (Dm6) were corrected to match *bam^1^*/*bam*^Δ86^ mutant flies, using a Q5 Site-Directed Mutagenesis Kit (New England Biosciences E0554S). Plasmids were injected into PhiC31 integrase transgenic flies and the constructs were integrated at attP40 on chromosome 2L, then selected for transformants by BestGene Inc. (Chino Hills, CA) (28).

### Immunofluorescence.

For whole mount preparations, whole testes were dissected in PBS, fixed in 4% paraformaldehyde (PFA) for 20 min and washed twice in PBS. The tissue was permeabilized in a solution of 1× PBS, 0.6% sodium deoxycholate, and 0.6% Triton for 1 h at room temperature, then washed twice before blocking overnight in PBST(Triton)-3% BSA (bovine serum albumin) at 4 °C. Primary antibodies were added at the following concentrations and testes were incubated rotating for two days: rabbit anti-HOW (1:50), mouse anti-Bam from DSHB (1:10), rabbit anti-Kmg (1:200) (14), mouse anti-HA (1:200), and goat anti-Vasa (1:100, Santa Cruz Biotechnology). After two PBS washes at room temperature, donkey secondary antibodies (Alexa Fluor, ThermoFisher) were added at 1:500 and the samples were incubated for 2 h at room temperature. Testes were then mounted on a slide in DAPI mount (Vectashield). To decrease background/nonspecific binding, the rabbit anti-HOW antibody (gifted by T. Volk) was preabsorbed with ~10 new wild-type testes overnight 3 times (fixed as for whole mount preparations.

For squashed preparations (samples stained with anti-How and anti-Bam), testes were placed on a SuperFrost Plus slide in a square drawn with a hydrophobic marker. Then the tissues were flattened live under a coverslip before being flash frozen in liquid nitrogen. After removing the coverslip, slides were incubated in cold 95% ethanol for 10 min before being fixed in 4% PFA for 7 min. Testes were washed in PBST (Triton-X) before being transferred to a wet chamber for blocking overnight in BSA at 4 °C, after which antibody staining continued as for whole mounts.

### EdU Labeling.

Cells in S phase were labeled with the Click-iT EdU Cell Proliferation Kit for Imaging–Alexa Fluor 555 dye (Invitrogen–C10338). Testes were dissected in Schneider’s (S2) medium and transferred into a drop of the same medium on a slide. The medium was then removed and replaced with 100 μM 5-ethynyl-2′-deoxyuridine (EdU) in S2 cell medium and incubated for 5 min at room temperature. Testes were then washed twice in S2 medium by removing the liquid with a pipette and replacing it with S2 medium. After the washes, testes were transferred with tweezers from the drop of S2 medium into a 1.7 mL Eppendorf tube with ~200 μL 1×PBS, then immediately the PBS was drawn off and replaced with 200 μL 4% paraformaldehyde in PBS for 20 min (rotating at room temperature) to fix, followed by two washes in PBST. The testes sheaths were then permeabilized in PBS with 0.6% Triton and 0.6% sodium deoxycholate for 1 h. The permeabilization mix was removed and the testes were rinsed once with PBST before adding the EdU detection reaction mix per the manufacturer’s instructions. Testes were then incubated in the dark with the reaction mix for 30 min, the reaction mix was removed, and the testes washed twice in 500 μL of PBST at room temperature. Blocking and antibody protocols continued in the same way as for squashes and whole mounts.

### HCR In Situs.

Probes to label the *how* coding sequence and the *how(L) 3’UTR* were designed following the method of Bedbrook et al. (29), which generated 44 probes (22 pairs) then ordered from Integrated DNA Technologies at 50 pmol/oligo. All other reagents were from Molecular Instruments (Los Angeles, CA), including H1 and H2 hairpins conjugated with 488. Testes were dissected in 1×PBS, fixed in 4% paraformaldehyde for 20 min, then permeabilized in 0.6% sodium deoxycholate for 30 min. Samples were then washed twice in PBS at RT.

HCR in situ hybridization followed the “sample in solution” HCR™ RNA-FISH protocol from Molecular Instruments with the following specifications: Probe solution was made with 1 μM of probes. Hybridization buffer and probe wash buffer were reduced to 200 μL from 500 and preamplification was done in 250 μL of amplification buffer. After completing the HCR protocol, DAPI mounting medium was added, testes were mounted on a slide and imaged by confocal microscopy.

### Imaging and Quantification.

Immunofluorescence stained or HCR FISH testes were imaged on a Leica SP8 Confocal microscope. Image brightness was adjusted in FIJI. To quantify overproliferation, testes were scored for the overall number of EdU positive cysts per testis and the number of EdU positive cysts with >16 cells each. Testes were also stained for the spermatocyte marker Kmg and scored for the presence of individual Kmg positive spermatocytes or spermatocyte cysts. For the quantification in Fig. 4, EdU and Kmg scoring was performed with the scorer blind to the genotypes.

### Analysis of Transcripts Expressed in Testes.

Microarray experiments were performed as described in ref. 14. To control for possible persistent changes in gene expression resulting from the heat shock treatment, control *bam^1^*/*bam*^Δ86^ flies lacking the *hsBam* transgene were subjected to 30 min of heat shock then cultured at 25 °C for 8, 16, 24, or 32 h in parallel with the experimental *hsBam; bam^1^*/*bam*^Δ86^ flies and the genes expressed in testes from the two genotypes were assessed by microarray in parallel. RNA-sequencing analysis was performed with RNA extracted from *hsBam; bam^1^*/*bam*^Δ86^ flies at each time point.

## Supplementary Material

Appendix 01 (PDF)

## Data Availability

RNA sequencing data have been deposited in the Gene Expression Omnibus (GSE274385 and GSE291938). Previously published data were used for this work (5).
