# Supplementary material for: An RNA-binding regulatory cascade controls the switch from proliferation to differentiation in the Drosophila male germ cell lineage
Source: Proc Natl Acad Sci U S A. 2025 May 16;122(20):e2418279122. doi: 10.1073/pnas.2418279122 (PMC12107169; doi:10.1073/pnas.2418279122)
Supplement: Supplementary file 1 — Appendix 01 (PDF) [file pnas.2418279122.sapp.pdf]

Supporting Information for

An RNA binding regulatory cascade controls the switch from proliferation to differentiation in the *Drosophila* male germ cell lineage

Devon E. Harris, Jongmin J. Kim, Sarah R. Stern, Hannah M. Vicars, Neuza R. Matias, Lorenzo Gallicchio, Catherine C. Baker, and Margaret T. Fuller#

Corresponding Author: Margaret T. Fuller#

Email: mtfuller@stanford.edu

This PDF file includes:

Supporting Text

Figures S1 to S6 with legends

Tables S1 and S2

SI References

Supporting Information

Methods

Microarray

RNA was extracted from about 30 pairs of dissected testes with seminal vesicles but without accessory glands using Trizol (Invitrogen). Reverse transcription was performed with oligo(dT)24 primer with a T7 promoter using ~200 ng of total RNA per Affymetrix protocol. The second strand was synthesized from the cDNA, and cRNA was produced by *in vitro* transcription. Fragmented cRNA was hybridized to the *Drosophila* genome 2.0 arrays (Affymetrix, Cat# 900532). All microarray experiments were performed by the Stanford Protein and Nucleic Acid facility. *how* transcript isoforms were distinguished by probes binding at the 3' end of transcripts, identified by Affymetrix Probeset ID (1637943_at). For analysis, all the raw CEL files were background adjusted and quantile normalized together by using R/BioConductor (v3.0.2) package GCRMA (1). Gene annotation was based on the Affymetrix file: "Drosophila_2.na32.annot.csv".

RNA sequencing

For analysis of transcript expression by RNA-Sequencing, 10~20 µg of total RNA was extracted from ~100 pairs of testes plus seminal vesicle from *hsBam; bam*^{1}/*bam*$^{\Delta 86}$ flies for each time point using Trizol (Invitrogen) followed by RNeasy cleanup (Qiagen) according to kit instructions. PolyA-tailed RNA was purified using the Oligotex mRNA kit (Qiagen, Cat#70022). Purified PolyA RNA was fragmented in the presence of random hexamer primers in first strand synthesis buffer (Invitrogen, Cat# 18080093) at 85˚C for 8 minutes. Fragmented RNAs were reverse transcribed using Superscript III reverse transcriptase (Invitrogen, Cat#

18080093) in the presence of RNaseOUT (Invitrogen Cat# 10777019) at 50˚C for one hour. From this step, we followed the directions in the NEBNext mRNA Library Prep Master Mix Set for Illumina (E6110s) to make libraries. DNA was purified using a QIAquick PCR purification kit (Qiagen Cat# 28104) after second strand synthesis, end repair, dA tailing, and adapter ligation. After adapter ligation, 300~500 bp fragments were size-selected by gel extraction (1.5% low-melt NUSIEVE gel in TBE). Pooled libraries were sequenced with Illumina HiSeq: 100bp, each paired-end with single indices.

For analysis of time course RNA-Seq data, raw fastq reads were trimmed using trim galore (version 0.4.3) to remove low-quality (Phred score 20) and adapter-containing (stringency 1) reads (2). Trimmed reads were mapped to the *Drosophila melanogaster* genome (BDGP6.46) with default parameters using STAR (version 2.5.3) (3). Mapped reads with quality scores smaller than 10 (-q 10) or not properly paired reads (-f 2) were discarded using SAMtools (version 1.4.1) (4). Counts per gene were obtained using the featureCounts function of the subread package (version 1.5.0) (5). Expression levels at different time points were TMM-normalized (Trimmed Mean of M-values), assuming the majority of housekeeping genes have the same expression levels in different time points using the R (version 4.1.0) package edgeR (version 3.34.0) (6). Axes for scatter plots were log2 transformed normalized CPM (Counts Per Million) + 1. Scatter plots were generated by Matlab (R2021a). Analyses scripts are available in: https://github.com/jongminkmg/HeldOutWings2024.

To ensure knockdown of *caf40,* we analyzed gene expression levels using RNA-seq for testes plus seminal vesicle from *CyO/+; bam*^{1}/*bam*$^{\Delta86}$ males (48hrs after heat shock) and *nosGal4* driven *caf40* knockdown flies (Figure S5). Testes plus seminal vesicle were dissected from 0–2 day old males in 1xPBS, as batches of 50 flies, in a cyclops dissecting dish for <30min at room

temperature. Each batch was transferred to a 1.7 ml Eppendorf tube containing 1ml of 1xPBS, the PBS was then immediately removed, and the testes were snap-frozen in liquid nitrogen and stored at −80°C. RNA was extracted from 150 pairs of testes using the RNeasy Plus Mini Kit from QIAGEN (74104). Frozen tissue was dissociated using a 1 ml syringe with a 27-gauge needle aspirating up and down ~10 times in 300μl of lysis buffer (from RNeasy kit) supplemented with 1:100 β-mercaptoethanol. Library preparation was carried out using the NEBNext® Ultra™ II Directional RNA Library Prep Kit for Illumina (#E7760S), with the NEBNext® Poly(A) mRNA Magnetic Isolation Module (#E7490S). Sequencing was performed by Novogene on a NOVAseq, PE 150 Illumina platform. Adapters and low-quality bases were trimmed with trimGalore and then aligned to the *Drosophila* dm6 genome using STAR. Differential expression analysis was performed using DEseq2.

Fertility tests

Fertility tests were performed with males from crosses that had been shifted to 29˚C for Gal4 driver expression. One male was placed in a vial with three virgin females and kept at 25˚C. Adults were removed after six days and vials were scored for the presence of pupa and adult offspring after at least 10 days.

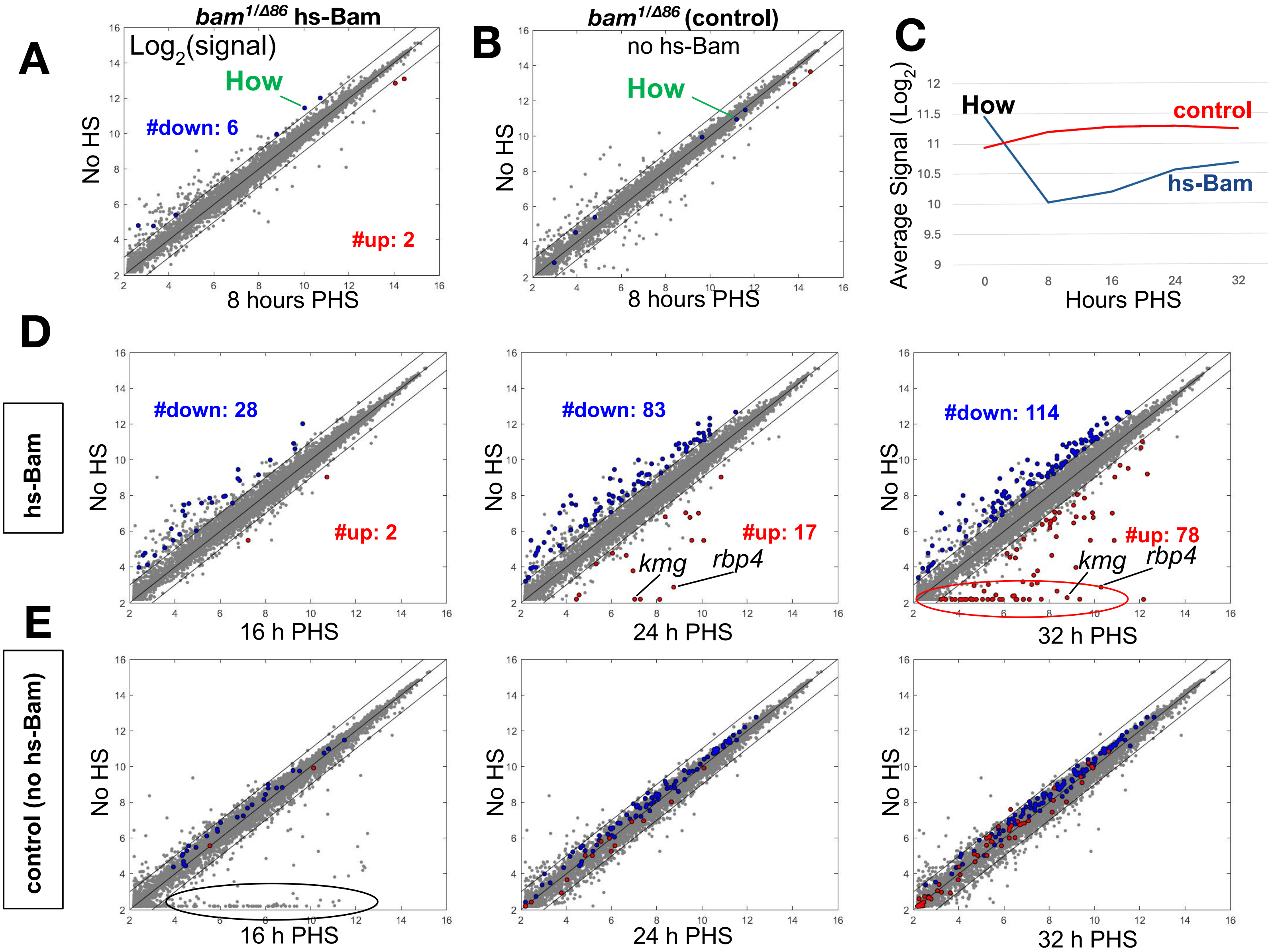

A
bam1/Δ86 hs-Bam
Log2(signal)
How
#down: 6
#up: 2
No HS
8 hours PHS
B
bam1/Δ86 (control)
no hs-Bam
How
No HS
8 hours PHS
C
How
control
hs-Bam
Average Signal (Log2)
Hours PHS
D
hs-Bam
#down: 28
#up: 2
No HS
16 h PHS
#down: 83
#up: 17
kmg
rbp4
24 h PHS
#down: 114
#up: 78
kmg
rbp4
32 h PHS
E
control (no hs-Bam)
No HS
16 h PHS
24 h PHS
32 h PHS

Figure S1. *how* is among the earliest transcripts to decrease after Bam is turned on.

(A, B) Scatterplots of transcript levels from microarray analysis comparing no heat shock and 8 hours PHS in (A) *bam*$^{1/\Delta 86}$; *hs-Bam* or (B) *bam*$^{1/\Delta 86}$ males lacking the *hs-Bam* construct, but subjected to the same heat shock regimen in parallel, to control for the effects of heat shock on gene expression. Genes colored blue (downregulated) or red (upregulated) were 1) not changed >2 fold in the microarray analysis of testes from control *bam*$^{1/\Delta 86}$ males lacking the *hs-Bam* construct but heat shocked then incubated for the indicated time and 2) also detected by independent RNA-seq as up or down >2 fold in *bam*$^{1/\Delta 86}$; *hs-Bam* males by RNA-sequencing at the time points indicated compared to testes from flies of the same genotype not subjected to heat shock. (C) Level of *how(L)* transcripts detected by microarray throughout the time course, showing decrease by 8h PHS in flies carrying the *hs-Bam* construct, but not in testes from control *bam*$^{1/\Delta 86}$ flies lacking the hs-Bam construct but subjected to the 30-minute pulse of incubation at high temperature, then shifted back to 25˚C as for the experimental genotype. (D, E) Microarray data from later time points after heat shock, showing the transcripts that increase in expression (red) and decrease in expression (blue) by the cutoff criteria (detected as up or down regulated >2 fold) in both this microarray comparison and in independent analysis by RNA-seq. (D) *bam*$^{1/\Delta 86}$; *hs-Bam* testes (red oval in *hs-Bam* 32h: genes expressed specifically in spermatocytes. (E) testes from control *bam*$^{1/\Delta 86}$ males that did not have the *hs-Bam* transgene, but were subjected to heat shock then incubated at 25°C for the indicated times. Black oval in (E) 16h PHS marks genes expressed in accessory glands that contaminated this sample.

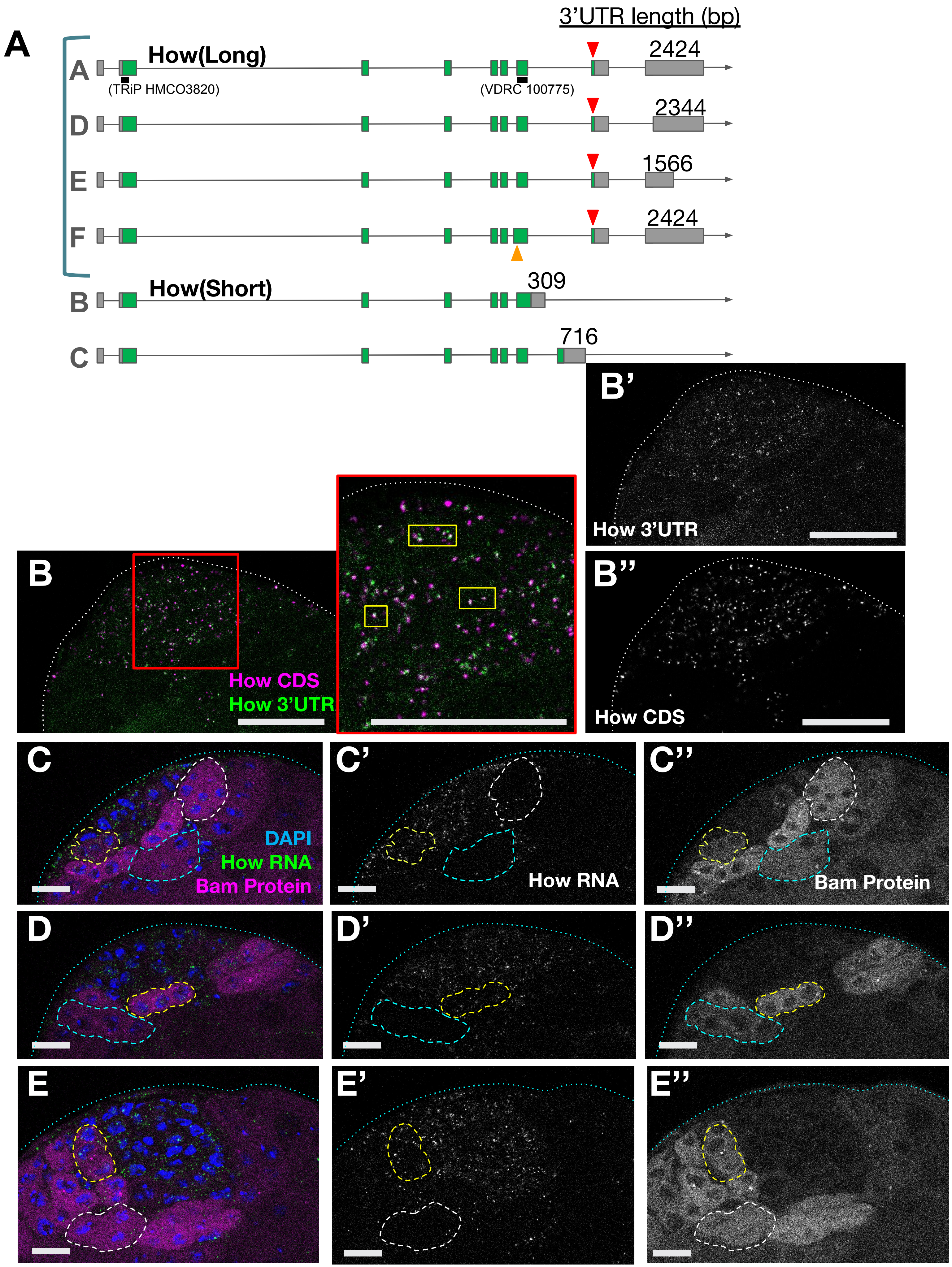
A
3'UTR length (bp)
How(Long)
A
(TRiP HMCO3820)
(VDRC 100775)
2424
D
2344
E
1566
F
2424
How(Short)
B
309
C
716
B'
How 3'UTR
B
How CDS
How 3'UTR
B''
How CDS
C
DAPI
How RNA
Bam Protein
C'
How RNA
C''
Bam Protein
D
D'
D''
E
E'
E''

Figure S2. How transcripts were detected in spermatogonia but not in spermatocytes

(A) Diagram of the *how* locus showing mRNA isoforms from FlyBase, designated A-F as in Flybase. The How(L) cDNA construct utilized in Figures 3 and 4 is RA and the How(S) cDNA construct utilized in Figure 3 is RB. Grey: UTRs. Green: protein coding sequence. Lines denote introns. Red arrowheads: nuclear localization signal. Orange arrowhead: additional coding sequence in isoform RF, distinguishing it from RA. Black bars: Sites of RNAi constructs utilized. (B) Apical tip of wild type testis showing distribution of *how* transcripts detected by Hybrid Chain Reaction (HCR FISH) using two different probe sets. (B) Merge. Magenta: Coding sequence (CDS) probe set; Green: 3'UTR probe set. Zoom in marked by red box. Yellow rectangles mark white loci of overlapping probes sets. (B') How(L) 3'UTR probe set. (B") Coding sequence (CDS) probe set. Scale Bar: 25 µm. (C-E) High magnification immunofluorescence images of apical tips of *Bam-GFP* testes with How protein coding sequence RNA labeled by HCR (additional examples for Figure 1G). Left: merge with (blue) DAPI, (green) *how* RNA, and (magenta) Bam-GFP. Middle: *how* RNA only. Right: Bam protein only. Yellow dashed outlines: early Bam positive cysts with *how* RNA present. White dashed outlines: later stage Bam positive cysts with fewer h*ow* RNA foci. Cyan dashed outlines: Bam positive cysts with no or very low *how* RNA signal detected. Scale bar: 12.5 µm.

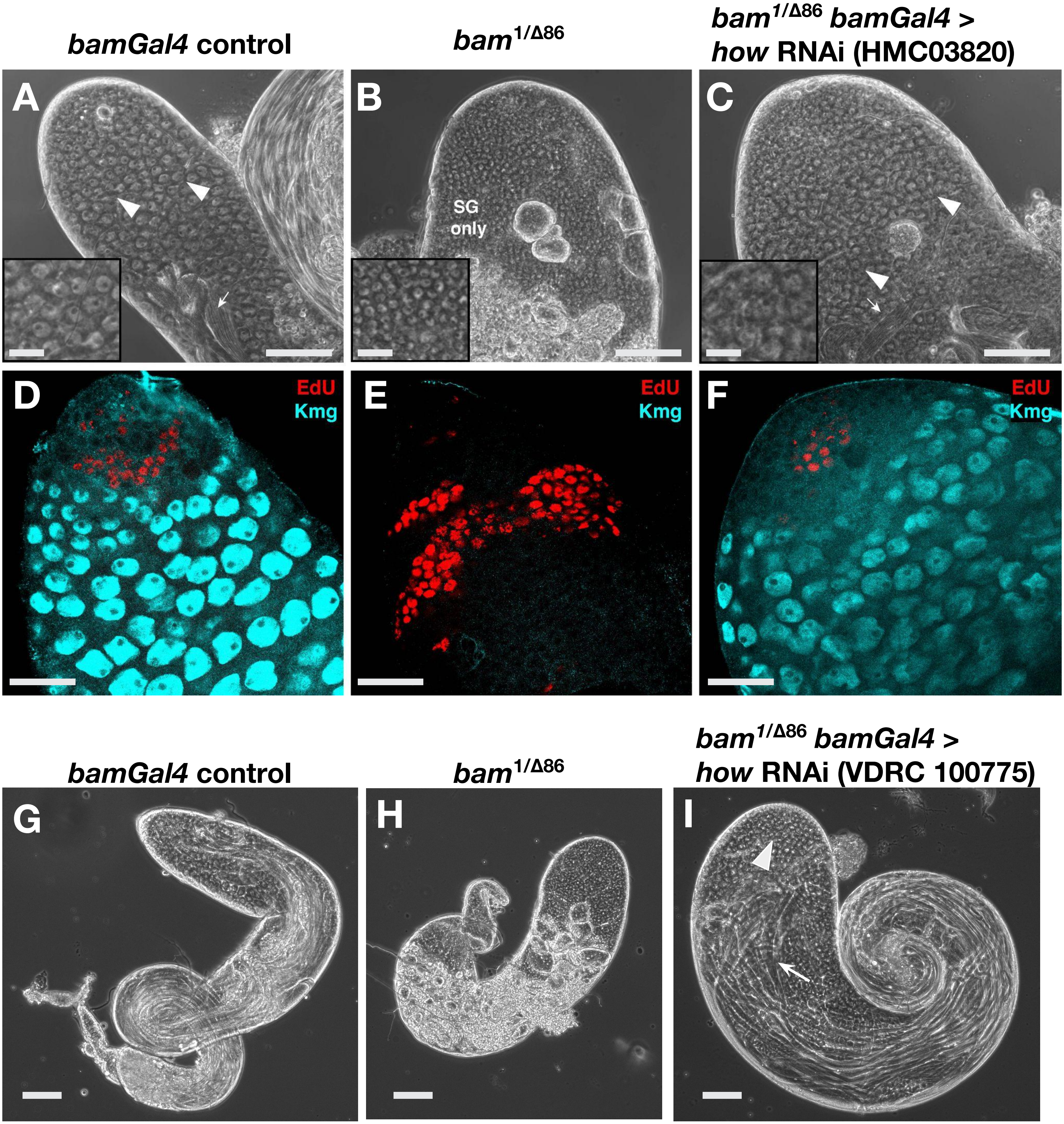

J

Genotype	Fertile Males
bamGal4	**4/6**
bam$^{1/\Delta 86}$	**0/3**
bam$^{1/\Delta 86}$ *bamGal4 >* *how* RNAi (VDRC 100775)	**5/9**

Figure S3. Knockdown of *how* in mid- to late spermatogonia in *bam* mutant males resulted in production of spermatocytes for two different RNAi lines.

(A-C) Phase contrast images of testis apical tips from (A) *bamGal4* driver only; (B) $bam^{1/\Delta 86}$; and (C) $bam^{1/\Delta 86}$; *bamGal4* > *how* RNAi (TRiP HMC03820 from Bloomington Stock #55665). Arrowheads: spermatocytes. Arrows: spermatid bundles. SG: spermatogonia. Scale bars: 100 µm, insets Scale Bars: 25 µm. (D-F) Immunofluorescence images of apical testis tips stained for EdU to mark S-phase nuclei (red) and Kmg to mark spermatocyte nuclei (blue). (D) Control: *bam*Gal4 driver only. (E) $bam^{1/\Delta 86}$. (F) $bam^{1/\Delta 86}$; *bam*Gal4 > *how* RNAi (TRiP HMC03820 from Bloomington Stock #55665). (G-I) Whole testis from (G) *bamGal4* driver only; (H) $bam^{1/\Delta 86}$; and (I) $bam^{1/\Delta 86}$; *bamGal4* > *how* RNAi (VDRC100775) for size comparison. Arrowhead: spermatocytes. Arrow: spermatid bundles. Scale bars: 100 µm. (J) Fertility tests of *bamGal4* driver only, $bam^{1/\Delta 86}$; and $bam^{1/\Delta 86}$; *bamGal4* > *how* RNAi (VDRC 100775) males. Males from all genotypes were progeny from crosses where the mated parents were allowed to lay eggs for 3 days at 25˚C then the parents were removed and the progeny shifted to 29˚C. Once the progeny males were collected, they were subjected to fertility tests at RT, ~ 25˚C.

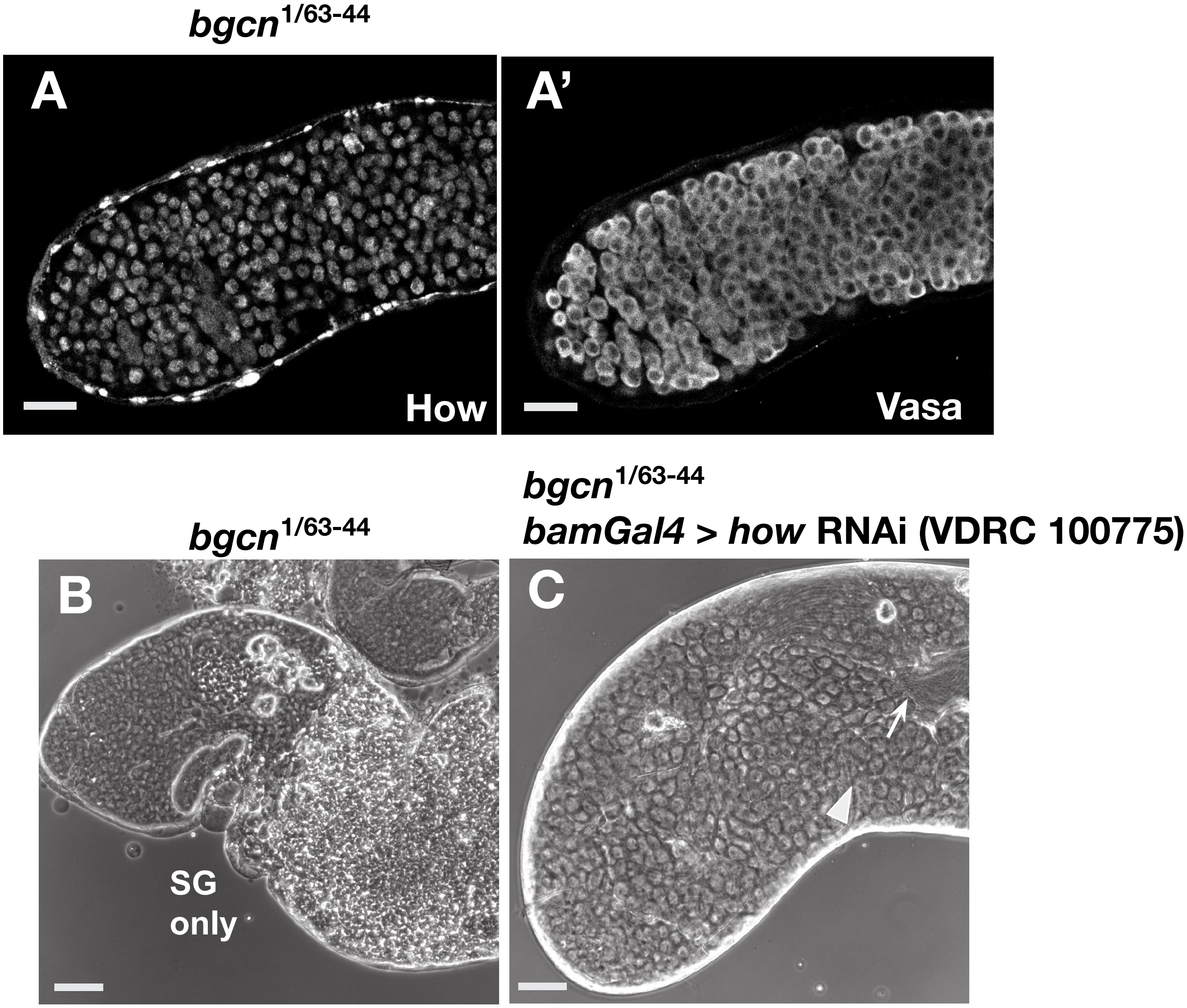

bgcn1/63-44
A
How
A'
Vasa
bgcn1/63-44
bgcn1/63-44
bamGal4 > how RNAi (VDRC 100775)
B
SG only
C

Figure S4: Knockdown of *how* in mid- to late spermatogonia in *bgcn* mutant males resulted in production of spermatocytes.

Immunofluorescence images of apical tip of *bgcn*$^{1/63\text{-}44}$ mutant testis stained with (A) anti-How and (A') anti-Vasa. Scale bars: 25 µm. (B,C) Phase contrast images of testis apical tips from (B) *bgcn*$^{1/63\text{-}44}$ versus (C) *bgcn*$^{1/63\text{-}44}$; *bam-Gal4* > *how RNAi* (VDRC 100775). Arrowhead: spermatocytes. Arrow: spermatid bundles. Scale bars: 50 µm.

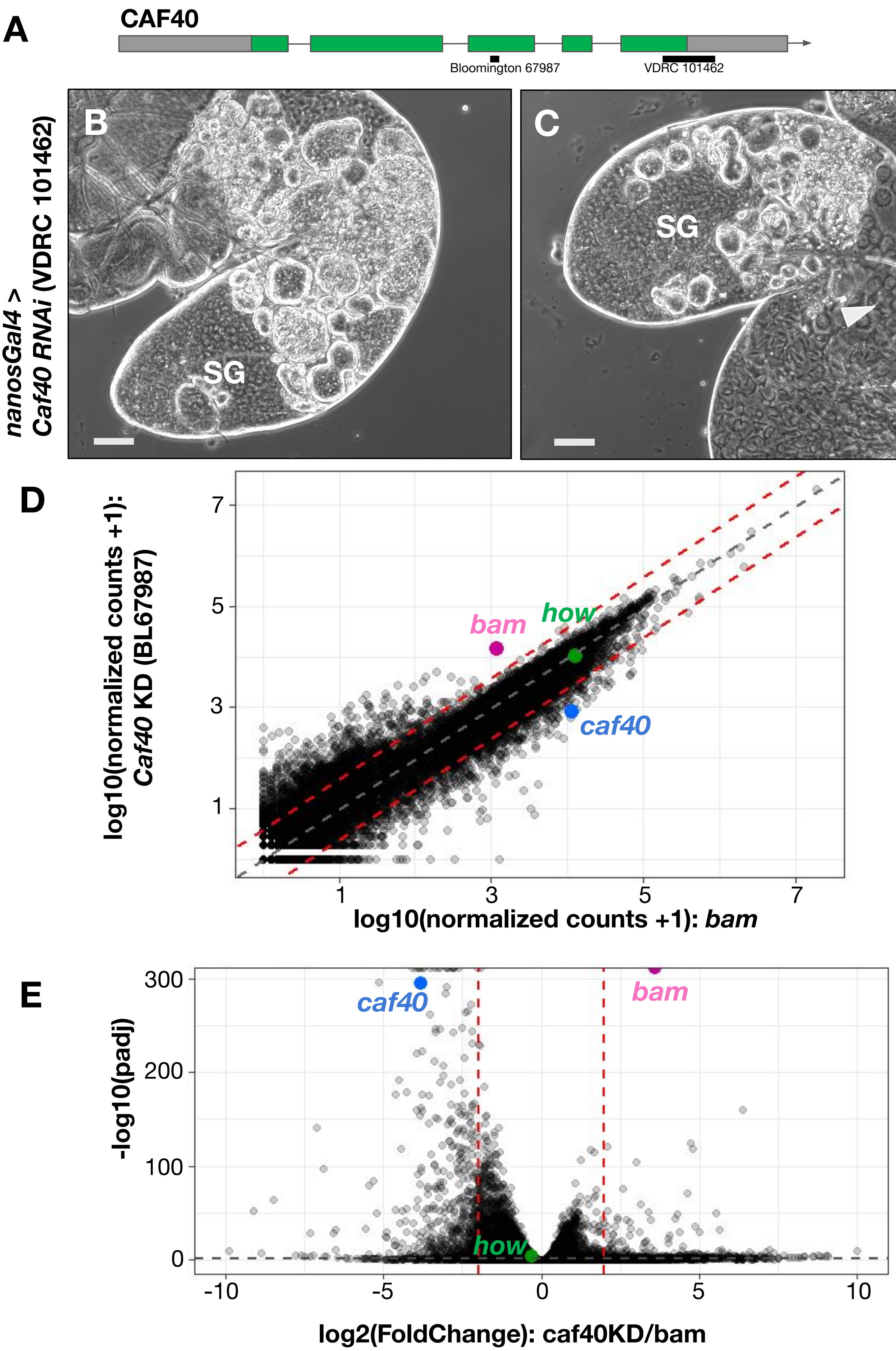

A
CAF40
Bloomington 67987
VDRC 101462
nanosGal4 >
Caf40 RNAi (VDRC 101462)
B
SG
C
SG
D
log10(normalized counts +1):
Caf40 KD (BL67987)
bam
how
caf40
1
3
5
7
log10(normalized counts +1): bam
E
-log10(padj)
300
200
100
0
caf40
bam
how
-10
-5
0
5
10
log2(FoldChange): caf40KD/bam

Figure S5. Knockdown of *caf40* in early germ cells by RNAi hairpin resulted in early germ cell overproliferation similar to *bam* mutants.

(A) Diagram of *Caf40* locus based on Flybase, showing sites of RNAi constructs tested (black lines). (B,C) Phase contrast images of testis apical regions showing two additional examples of *nosGal4 VP16* driving knockdown of *Caf40* using a different *RNAi* line (VDRC 101462). Although the knockdown phenotype was not as strong as for the RNAi construct carried in Bloomington Stock #67987 (Figure 5), there was still overproliferation of small early germ cells followed by cell death. SG: spermatogonia. Arrowhead: spermatocytes. Scale bars: 50 µm. (D,E) Comparison of gene expression levels in testes from *nosGal4;UASCaf40 RNAi* (Bloomington 67987) *versus* $bam^{1/\Delta 86}$ males 48hrs after heat shock. D) RNA-Seq data showing expression levels per gene plotted as log10(normalized counts + 1). E) Volcano plot comparing RNA-Seq data from (D). (Blue dot) expression of *Caf40*: RNA-Seq confirmed that the knockdown lowered levels of *Caf40* RNA by approximately 14-fold. (Magenta dot) expression of *bam*: The level of *bam* mRNA detected was higher in *Caf40* knockdown than in *bam* mutant testes, likely in part because $bam^{\Delta 86}$ deletes most of the *bam* coding sequence (26). (Green dot) expression of *how*: RNA-Seq confirmed that levels of *how* RNA in *nosGal4;UASCaf40 RNAi* testes were similar to the levels in *bam* mutant testes.

A

bamGal4 > how RNAi
VDRC 100775

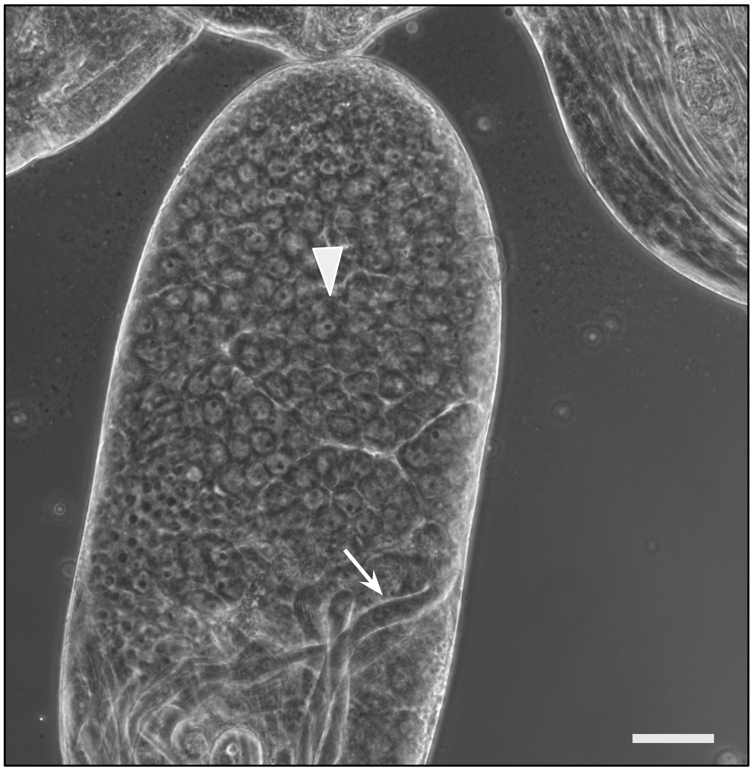

B

bamGal4> how RNAi
TRiP HMC03820
Bloomington 55665

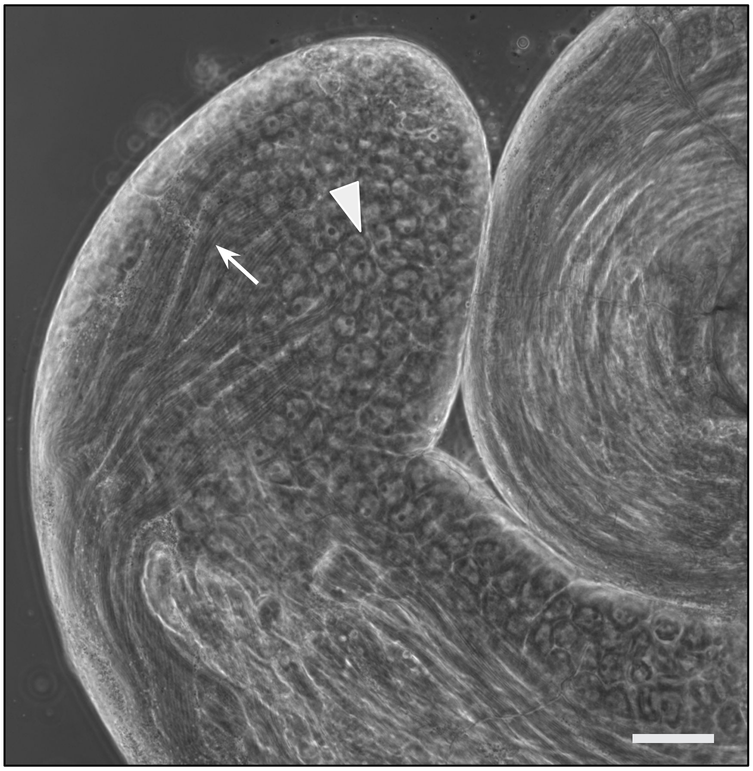

Figure S6. Knockdown of *how* in wild-type mid to late spermatogonia did not affect the switch to differentiation.

(A,B) Phase contrast images of testis apical regions from flies expressing different *how* RNAi hairpins driven by *bamGal4*: (A) Vienna Drosophila Resource Center (VDRC)100775. (B) TRiP HMC03820 from Bloomington Drosophila Stock #55665. Arrowheads: spermatocytes. Arrows: elongating spermatids. Scale bars: 50 µm.

Table S1. *how* coding sequence HCR probes

GTCCCTGCCTCTATATCTTTCCTGGGCGGACAATATGCTGGC
GGTCGAAGGCGGCTGTCGGAGCTTCCACTCAACTTTAACCCG
GTCCCTGCCTCTATATCTTTGGGATTCAGGATCAATGGGGCG
CGCCGTTGTGGGGACGGTCATCTTCCACTCAACTTTAACCCG
GTCCCTGCCTCTATATCTTTCGGATCTGGGCGGCAAGGCCGG
CCAAGCGGGGCGGCGGCGGGTGTTCCACTCAACTTTAACCCG
GTCCCTGCCTCTATATCTTTGGCTATCAGAGGCGGCAACCAG
GCAGGCCGGTGGATGTCAGCAGTTCCACTCAACTTTAACCCG
GTCCCTGCCTCTATATCTTTTGCGACAGATTTCGCTGTTGTG
GCGGCGCCACTCCTCATCGCACTTCCACTCAACTTTAACCCG
GTCCCTGCCTCTATATCTTTGCCAATTCCATGAGTTGACGTT
TCCCTATAAGTGCCATTAATAATTCCACTCAACTTTAACCCG
GTCCCTGCCTCTATATCTTTCTTGCGGCACGAGCAACTTCTG
TCTTTAGCTCATCTTCGCCTTCTTCCACTCAACTTTAACCCG
GTCCCTGCCTCTATATCTTTCTTCACTGTGGCACGGTTCTCG
TACTTCGGCGACGGCCTGGGCCTTCCACTCAACTTTAACCCG
GTCCCTGCCTCTATATCTTTTGCAGGTCATCGGACAGATGCT
GTGTCCTCGACGGTTATCAGGATTCCACTCAACTTTAACCCG
GTCCCTGCCTCTATATCTTTCGTCCTCCTTCTTCTTGTCGCG
CCCAGTTAGGCTTGCCACGGTTTTCCACTCAACTTTAACCCG
GTCCCTGCCTCTATATCTTTAATCTTGCAGCCGGTCTCCTGT
CATGGAACCCTTGCCTCGGACCTTCCACTCAACTTTAACCCG
GTCCCTGCCTCTATATCTTTCGGGGTCCCAAAATGCGACCGA
TCCAATTGCTTGGCGGTCATGCTTCCACTCAACTTTAACCCG
GTCCCTGCCTCTATATCTTTGGACTGGCACATAAACCTTCTC
CAAAGTTGAAATCTGGATGCTCTTCCACTCAACTTTAACCCG
GTCCCTGCCTCTATATCTTTGGGTTCGGGCAGAGTGAGCGGC
GTTCATCGTCACCACAGAGCCCTTCCACTCAACTTTAACCCG
GTCCCTGCCTCTATATCTTTAACAGTGAGGCGCGCACGCGTG
TCCTTCTTGACCCCATTGATCTTTCCACTCAACTTTAACCCG
GTCCCTGCCTCTATATCTTTCGACGTGGGTGAAGACGTTGGG
CAATTTCTTCGTCCAGCAGGCGTTCCACTCAACTTTAACCCG
GTCCCTGCCTCTATATCTTTCTTGAGCAACTGGGCCAGATAG

Table S1. continued

GAAGGCGGCCAGTTGCTTGCGGTTCCACTCAACTTTAACCCG
GTCCCTGCCTCTATATCTTTTGCTGCTGCTGTGGGGTCAAGT
TCGGCGATGCTCTGTGTGCTCTTTCCACTCAACTTTAACCCG
GTCCCTGCCTCTATATCTTTCCTGCGGCGCCTGTTGCTGCTG
GCTGCGGGGTCATGGGGACCACTTCCACTCAACTTTAACCCG
GTCCCTGCCTCTATATCTTTTTGGGCTTGAGCCTGCTGCTGT
CTGTGCCTGAGCCTGAGCCTGGTTCCACTCAACTTTAACCCG
GTCCCTGCCTCTATATCTTTGCTGCCTGTTGCTGCAAGTGCT
TGCGCGACCGCAACAACTGCTGTTCCACTCAACTTTAACCCG
GTCCCTGCCTCTATATCTTTCTTTGCTCTCACAGACACTCAT
GCTGCAGTTGCTGTTGCACAACTTCCACTCAACTTTAACCCG

Table S2. *how(L)* 3’UTR HCR probes

CTCACTCCCAATCTCTATAATATATTTAAATATATATATATA
CTTGTATATGCATTTGGATGTAAACTACCCTACAAATCCAAT
CTCACTCCCAATCTCTATAATTTTGTTCGTTGAACTTTTACA
TGTATATGCTTGCATTAAGTATAACTACCCTACAAATCCAAT
CTCACTCCCAATCTCTATAATTAGATTATCATAATTAAACTA
CTAAGAACTAACTTGTATTTTCAACTACCCTACAAATCCAAT
CTCACTCCCAATCTCTATAAAATCGTTTGCTTAATTGCCTTG
AGCTCTATGTTTGATTCTCTTAAACTACCCTACAAATCCAAT
CTCACTCCCAATCTCTATAAAGTTTAATGGCCACAAGATTCA
TTTTGTTTGCTGGCAAAACGTAAACTACCCTACAAATCCAAT
CTCACTCCCAATCTCTATAACGTTTGCTGTTGCTGTTGCTTT
ATACATACTGATGTTGCTGCTGAACTACCCTACAAATCCAAT
CTCACTCCCAATCTCTATAATGTGATGCTGCTGGTGTTGAAT
TGCTTTTGCTTTTGCTGTTGATAACTACCCTACAAATCCAAT
CTCACTCCCAATCTCTATAACAAATAGGTTCAGAGTTTGGAG
GTGCCGCATCTGCTGCTCATTTAACTACCCTACAAATCCAAT
CTCACTCCCAATCTCTATAATTTTAGTAGTTGTGTAAGTGAC
TTTGGCGTTCGGAGGGGTTCAGAACTACCCTACAAATCCAAT
CTCACTCCCAATCTCTATAATTTTGGTTACGCTTTTTTGTTA
ATGTGGTGGCTCAGGATATCTGAACTACCCTACAAATCCAAT
CTCACTCCCAATCTCTATAAACACACGTGGCTCTGCTTTCGG
GCTTCAAGTAAGCACATTCACAAACTACCCTACAAATCCAAT
CTCACTCCCAATCTCTATAACTGATTCCGTGCTCATCTACGG
CCTGGCTGGGATGCTGCTGCTGAACTACCCTACAAATCCAAT
CTCACTCCCAATCTCTATAAGCGGATGTGGGAGCGGATTGGT
CAGGCGATGGATTCATGGTTCAAACTACCCTACAAATCCAAT
CTCACTCCCAATCTCTATAATTTCCTGTGGAGGCGGTGCGCT
AGTGTGTTTGGGACATTTGTTTAACTACCCTACAAATCCAAT
CTCACTCCCAATCTCTATAAATCAGGGCAGAGCGGCCAGGCC
TGAAAATTCTTTTTCAGAACCCAACTACCCTACAAATCCAAT
CTCACTCCCAATCTCTATAACATCGAACATACAGTGTGGATT
AGGCCAACGATTCCGGTCAGGTAACTACCCTACAAATCCAAT
CTCACTCCCAATCTCTATAAGCAGCATTTGTATCAAAATAAA
GGAATTGGGGATGGTCAGTTATAACTACCCTACAAATCCAAT
CTCACTCCCAATCTCTATAACTAAAGTGATCGGCTGCCTGGC

Table S2. continued

TAGCAAAATTTTAAAATAATTGAACTACCCTACAAATCCAAT
CTCACTCCCAATCTCTATAACGCAATCGTTCGTTATCCGTTG
GTACGCAATCAAAGGGCAGGCAAACTACCCTACAAATCCAAT
CTCACTCCCAATCTCTATAATCGTACATTATTGATTTAAAGT
TTCTTAATGGTTTCTTTTCTTCAACTACCCTACAAATCCAAT
CTCACTCCCAATCTCTATAAGTGTGATTTTAGAGTGTTACTT
TATAAAGTTGCTTTCGCCGATAAACTACCCTACAAATCCAAT
CTCACTCCCAATCTCTATAACTCGATTCACTATGCATGGTAT
TCTGAGTTATAGTGCTTCGAATAACTACCCTACAAATCCAAT

SI References

1. B. M. Bolstad, R. A. Irizarry, M. Astrand, T. P. Speed, A comparison of normalization methods for high density oligonucleotide array data based on variance and bias. *Bioinforma. Oxf. Engl.* **19**, 185–193 (2003).

2. M. Martin, Cutadapt removes adapter sequences from high-throughput sequencing reads. *EMBnet.journal* **17**, 10–12 (2011).

3. A. Dobin, *et al.*, STAR: ultrafast universal RNA-seq aligner. *Bioinforma. Oxf. Engl.* **29**, 15–21 (2013).

4. H. Li, *et al.*, The Sequence Alignment/Map format and SAMtools. *Bioinforma. Oxf. Engl.* **25**, 2078–2079 (2009).

5. Y. Liao, G. K. Smyth, W. Shi, featureCounts: an efficient general purpose program for assigning sequence reads to genomic features. *Bioinforma. Oxf. Engl.* **30**, 923–930 (2014).

6. M. D. Robinson, D. J. McCarthy, G. K. Smyth, edgeR: a Bioconductor package for differential expression analysis of digital gene expression data. Bioinforma. Oxf. Engl. 26, 139–140 (2010).
